# Design, Synthesis and Molecular Docking of Novel Acetophenone-1,2,3-Triazoles Containing Compounds as Potent Enoyl-Acyl Carrier Protein Reductase (InhA) Inhibitors

**DOI:** 10.3390/ph15070799

**Published:** 2022-06-27

**Authors:** Fawzia Faleh Albelwi, Hanaa M. Abdu Mansour, Maram M. Elshatanofy, Yeldez El Kilany, Kamal Kandeel, Bassma H. Elwakil, Mohamed Hagar, Mohamed Reda Aouad, El Sayed H. El Ashry, Nadjet Rezki, Maged A. El Sawy

**Affiliations:** 1 Department of Chemistry, Faculty of Science, Taibah University, Al-Madinah Al-Munawarah 30002, Saudi Arabia; ffs.chem334@gmail.com (F.F.A.); hana.m.a.mansoor@gmail.com (H.M.A.M.); aouadmohamedreda@yahoo.fr (M.R.A.); 2 Department of Chemistry, Faculty of Science, Alexandria University, Alexandria 21321, Egypt; melshatanofy@gmail.com (M.M.E.); yeldez244@yahoo.com (Y.E.K.); mohamedhaggar@gmail.com (M.H.); eelashry60@hotmail.com (E.S.H.E.A.); 3 Department of Biochemistry, Faculty of Science, Alexandria University, Moharam Beik, Alexandria 21547, Egypt; kamkandeel@yaho.com; 4 Department of Medical Laboratory Technology, Faculty of Applied Health Sciences Technology, Pharos University in Alexandria, Alexandria 21311, Egypt; bassma.hassan@pua.edu.eg; 5 Department of Pharmaceutical Chemistry, Faculty of Pharmacy, Pharos University, Alexandria 21311, Egypt; maged.elsawy@pua.edu.eg

**Keywords:** 1,2,3-triazole, CuAAC, molecular modeling, InhA

## Abstract

New medications are desperately needed to combat rising drug resistance among tuberculosis (TB) patients. New agents should ideally work through unique targets to avoid being hampered by preexisting clinical resistance to existing treatments. The enoyl-acyl carrier protein reductase InhA of *M. tuberculosis* is one of the most crucial targets since it is a promising target that has undergone extensive research for anti-tuberculosis drug development. A well-known scaffold for a variety of biological activities, including antitubercular activity, is the molecular linkage of a1,2,3-triazole with an acetamide group. As a result, in the current study, which was aided by ligand-based molecular modeling investigations, 1,2,3-triazolesweredesigned and synthesized adopting the CuAAC aided cycloaddition of 1-(4-(prop-2-yn-1-yloxy)phenyl)ethanone with appropriate acetamide azides. Standard spectroscopic methods were used to characterize the newly synthesized compounds. In vitro testing of the proposed compounds against the InhA enzyme was performed. All the synthesized inhibitors completely inhibited the InhA enzyme at a concentration of **10** µM that exceeded Rifampicin in terms of activity. Compounds **9**, **10**, and **14** were the most promising InhA inhibitors, with IC_50_ values of 0.005, 0.008, and 0.002 µM, respectively. To promote antitubercular action and investigate the binding manner of the screened compounds with the target InhA enzyme’s binding site, a molecular docking study was conducted.

## 1. Introduction

Triazoles, a class of nitrogen-containing heterocycle, are well known as intriguing structural motifs with fascinating applications in many areas of disciplines such as materials research, synthetic organic chemistry, and drug discovery [1].

Due to their well-documented therapeutic potential, 1,2,3-triazole analogues are attractive target substances for researchers worldwide. They are important antiviral [2,3], antibacterial [4,5,6,7], antifungal [8,9], antimalarial [10,11], anticancer [12,13,14,15,16,17,18], analgesic [11], antihypertensive [12], anticonvulsant [19], and CNS depressing [20] agents.

Furthermore, as enticing linker units suitable for connecting two pharmacophores in order to develop novel polyfunctional drugs, 1,2,3-triazoles have become increasingly relevant and important in the generation of bioactive and multifunctional drugs [21]. Due to the obvious polarity of the core of 1,2,3-triazoles, they induce non-covalent bonding interactions with microbial proteins, such as dipole–dipole and pi-stacking, which restricts their development, resulting in a potent antibacterial drug [22,23].

**Figure 1:**
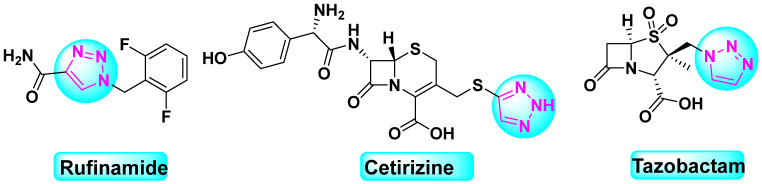
Drugs with the 1,2,3-triazole unit on the market.

Moreover, several drugs on the market, such as rufinamide (anticonvulsant), cetirizine (antibiotic), and tazobactam (antibacterial agent) have inserted a 1,2,3 triazole core into their framework [24] (Figure 1).

**Figure 2:**
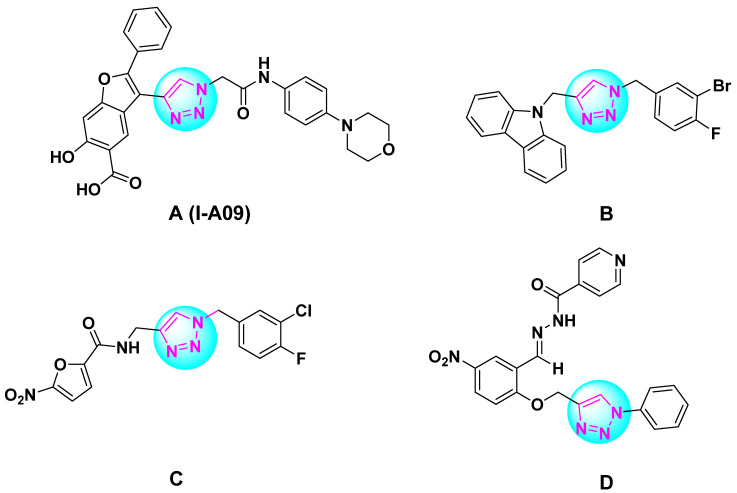
Representative examples of 1,2,3-triazole derivatives having antitubercular activity.

**Figure 3:**
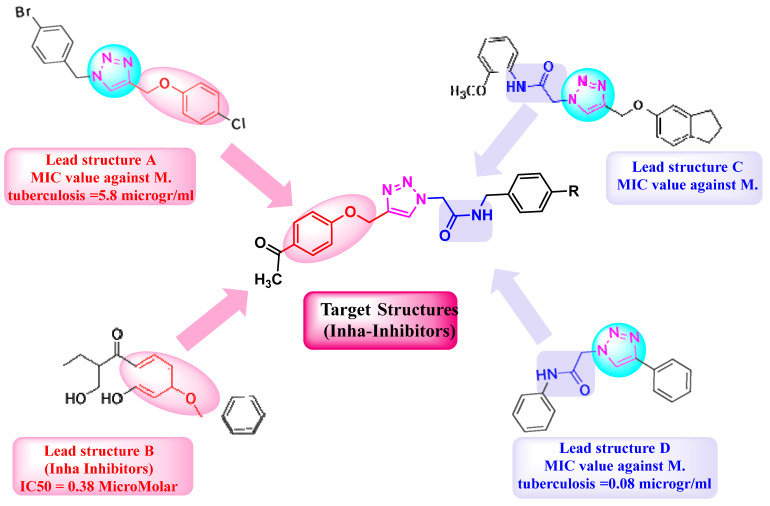
Ligand-based design of target structure.

In the literature, there are numerous 1,2,3-triazoles tethered to bioactive heterocyclic moieties, such as (Figure 2A–D), that have been documented as promising antitubercular agents [25,26,27,28,29,30,31]. In particular, the 1,4-disubstituted 1,2,3-triazole derivatives were identified and proven to have high efficacy against Mycobacterium tuberculosis. For instance, lead structure A with MIC = 5.8 μg/mL was constructed from a triazole scaffold substituted at 1-position by a 4-bromobenzyl moiety and at 4-position by a 4-chlorophenoxymethyl moiety [32] (Figure 3). The importance of the phenoxy methyl moiety in the development of the new candidate with significant antimycobacterium activity is represented by the lead structure (Figure 3B), which exerts its activity through inhibition of the enoyl-acyl carrier protein reductase InhA enzyme, IC_50_ = 0.38 μM [33].

On the other hand, different aryl derivatives can be linked to the 1,2,3-triazole scaffold via an acetamide linker, as shown by lead structures (Figure 3C,D), which have MICs of 6.25 and 0.08 g/mL, respectively, and have shown good antitubercular activity [34,35].

In light of these facts, and as a follow-up to our efforts on the synthesis of such bioactive scaffolds [1,2,3,4,5,6,7,8,9,10,11,12,13,14,15,16,17,18], we have anticipated to design and synthesize a novel 1,4-disubstituted 1,2,3-triazolecontainingacetamide in order to link aryl derivatives to the triazole motif and an acetyl phenoxy methyl moiety substituting the 4-position of the triazole ring. The resulting 1,2,3-triazole adducts were investigated as inhibitors of the NADH-dependent enoyl-acyl carrier protein reductase enzyme. InhA contributes to the manufacture of key components of the mycobacterial cell walls through involvement in the type II fatty acid biosynthetic pathway. Among the studied compounds, one demonstrated good inhibitory activity against the InhA enzyme with an IC_50_ of 0.002 μM and was considered the most active molecule. Molecular modeling was also used to evaluate the pattern of binding of the tested drugs to the target enzyme.

## 2. Results and Discussion

### 2.1. Chemistry

**Scheme 1:**
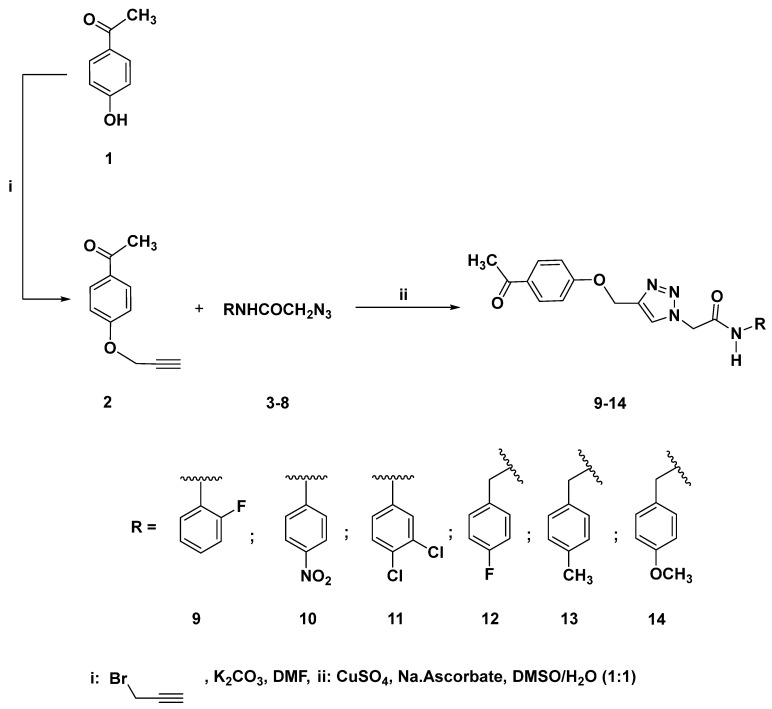
Click synthesis of 1,2,3-triazole-acetophenone conjugates **9**–**14**.

The propargylation of 4-hydroxyacetophenone (**1**) with 3-bromoprop-1-yne has been carried out in dimethylformamide as a solvent and in the presence of a catalytic amount of potassium carbonate as the basic catalyst, which resulted in the formation of precursor 1-(4-(prop-2-yn-1-yloxy) phenyl)4thenone (**2**) in 91% yield, Scheme 1.

The formation of *O*-propargylated acetophenone **2** as the key intermediate has been inferred from its spectral information (IR, ^1^H NMR and ^13^C NMR).

The IR spectrum unveiled a significant characteristic absorption bands, confirming the proposed structure. Sharp bands at 2100 and 3210 cm^−1^ were detected, which were ascribed to the acetylenic hydrogen carbons (C≡C) and (≡C-H) groups, respectively. In addition, the vanishing of the hydroxyl absorption band proved that it had been propargylated.

Examination of ^1^H and ^13^C NMR spectra of alkyne showed the existence of an alkyne side chain; based on the presence of a diagnostic doublet at δ_H_ 4.77 ppm attributed to OCH_2_ protons. The ≡C-H proton resonated as a broad singlet at δ_H_ 2.57 ppm overlapping with the signal containing three protons of the methyl group. The aromatic protons resonated in the aromatic area as expected (δ_H_ 7.03–7.97 ppm).

In contrast, the existence of significant propargyl carbon signals (OCH_2_ and C≡C) at δ_C_ 51.08, 71.48 and 73.00 ppm was also compatible with such acetylenic side chains. The signals recorded at δ_C_ 109.68–156.51 ppm were assigned to the aromatic carbons of the phenyl ring. The carbonyl carbon was also observed in the downfield area at δ_C_ 192.09 ppm.

The *O*-propargylated acetophenone **2** is a versatile key intermediate for the click synthesis of novel 1,2,3-triazoles-based acetophenone **9**–**14**. Thus, 1,3-dipolar cycloaddition of the synthesized alkyne **2** with some appropriate phenyl **3**–**5** and/or benzyl acetamide azides **6**–**8** afforded the corresponding 1,4-disubstituted 1,2,3-triazoles **9**–**14** in very good yields (87–90%), Scheme 1. The click reactions were carried out at room temperature for 6–8 h in the presence of a mixture of dimethylsulfoxide and water (DMSO/H_2_O; 1:1) as solvents and catalytic amount of copper sulfate and Na ascorbate as catalysts as described in Scheme 1.

The combination of IR, ^1^H NMR, and ^13^C NMR spectral analysis has verified the formation of triazoles **9**–**14**. Compound **9** has been used as a model in order to assign, absorption bands in the IR spectra and resonance peaks in the NMR spectra.

Triazole 9 has an IR spectrum that matches its assigned structure. The disappearance of the peaks belonging to C≡C and ≡C-H absorption bands and the appearance of a sharp absorption at 3300 cm^−1^ attributed to the NH group of the acetamide linkage indicates in part that the cycloaddition reaction has taken place.

The ^1^H NMR spectrum of triazole **9** reveals the disappearance of the signal attributed to the ≡C-H proton of its precursor *O*-alkyne **2** and the appearance of a diagnostic singlet at δ_H_ 8.25 ppm, assigned to the CH-1,2,3-triazole proton. The spectrum also shows two pairs of singlets for the OCH_2_ and NCH_2_ protons at δ_H_5.24 and 5.41 ppm, respectively. Moreover, the assigned CONH proton at δ_H_ 10.29 ppm was also in agreement with the assigned 1,2,3-triazole **9**. Further assignment of the aromatic protons revealed that they were resonated at their appropriate positions and listed in the experimental section.

In addition, the ^13^C NMR spectrum of compound **9** also revealed the absence of the two sp-carbons and the presence of OCH_2_ and N**C**H_2_-carbons at δ_C_ 52.96 and δ_C_ 61.74 ppm, respectively. A new signal also appeared at δ_C_165.23 assigned to the carbonyl amide carbon (CONH). All sp^2^ carbons were observed in the aromatic region (See experimental section).

On the other hand, the click synthesis has also been evidenced by the ^19^F NMR spectrum of compound 9, which showed multiple signals recorded between δ_F_ −124.75 to −124.69 ppm, which confirmed the presence of an aromatic fluorine atom in the structure.

### 2.2. Biological Evaluation

**Figure 4:**
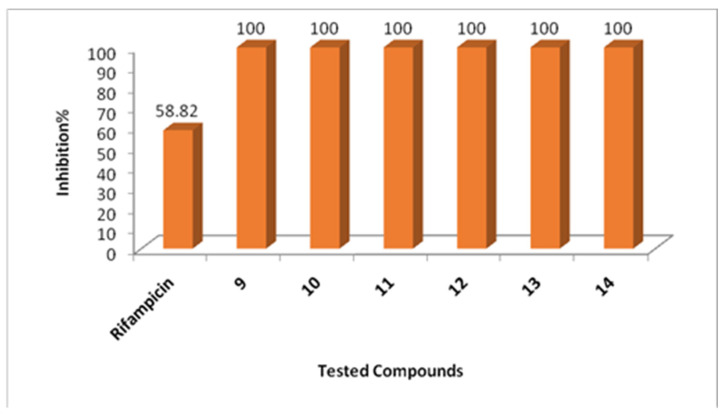
The inhibition percentage at **10** µM.

A brief screening of the synthesized compounds was tested as a direct InhA enzyme inhibitor. The InhA inhibition (IC_50_) was calculated (Table 1). All synthesized inhibitors completely inhibited the InhA enzyme at a concentration of 10 µM (Figure 4), excelling in terms of activity, with a rifampicin IC_50_ of value 8.5 µM. Moreover, all synthesized inhibitors showed activity better than isoniazid IC_50_ of value 0.054 µM except compound 13 IC_50_ of value 0.084 µM [36]. Furthermore, 9,10, and 14 were the most promising InhA inhibitors, with IC_50_ values of 0.005, 0.008, and 0.002 µM, respectively.

## 3. Molecular Modeling

### Docking Simulations

All synthesized compounds are subjected to molecular docking studies into the binding site of inhA in order to understand hypothesized intermolecular interactions and consider the potential binding pattern that supports these drugs’ inhibitory effects. Molecular operating environment software was used to accomplish this (MOE 2016.0802). The protein data bank provided the X-ray crystal structures of InhA (PDB ID: 4UVD) with its co-crystallized ligand (HRW) (PDB DOI: 10.2210/pdb4UVG/pdb).

The docking poses were chosen based on the top-scored conformation with the best binding interactions found by the MOE search algorithm and scoring function. Furthermore, binding energy scores, creation of binding interactions with neighboring amino acid residues, and relative placement of docked poses in respect to co-crystallized ligands all played a role in defining binding affinities to the enzyme binding pockets and docking to the InhA active site.

**Figure 5:**
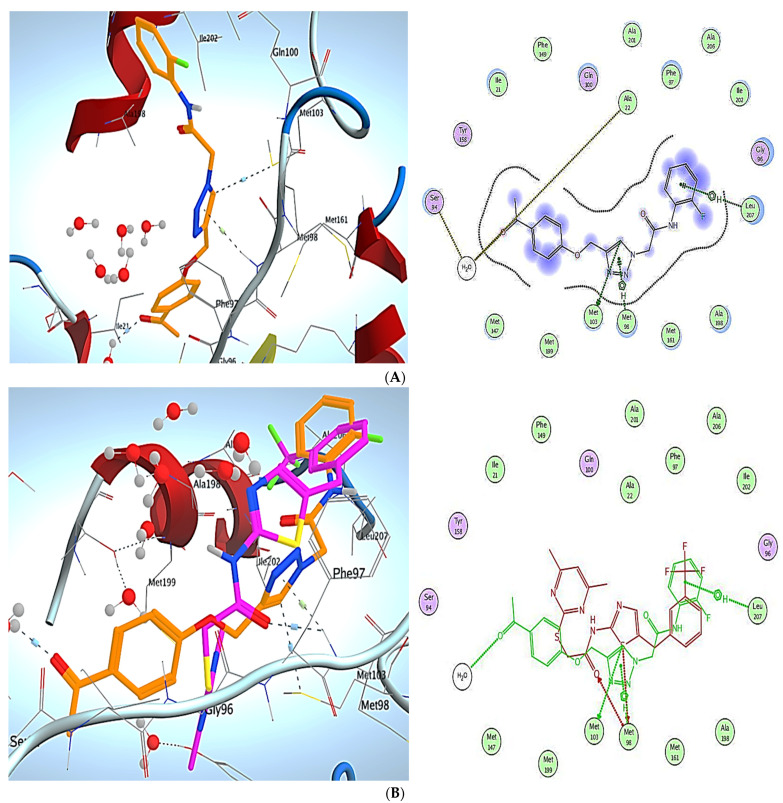
(**A**): Docking and binding pattern of compound **9** into InhA active site (PDB ID: 4UVD) in 2D (**right panel**) and 3D (**left panel**). (**B**): An overlay of the docked pose of compound **9** (brown) with the co-crystallized ligand (purple) into inhA active site in 2D (**right panel**) and 3D (**left panel**).

**Figure 6:**
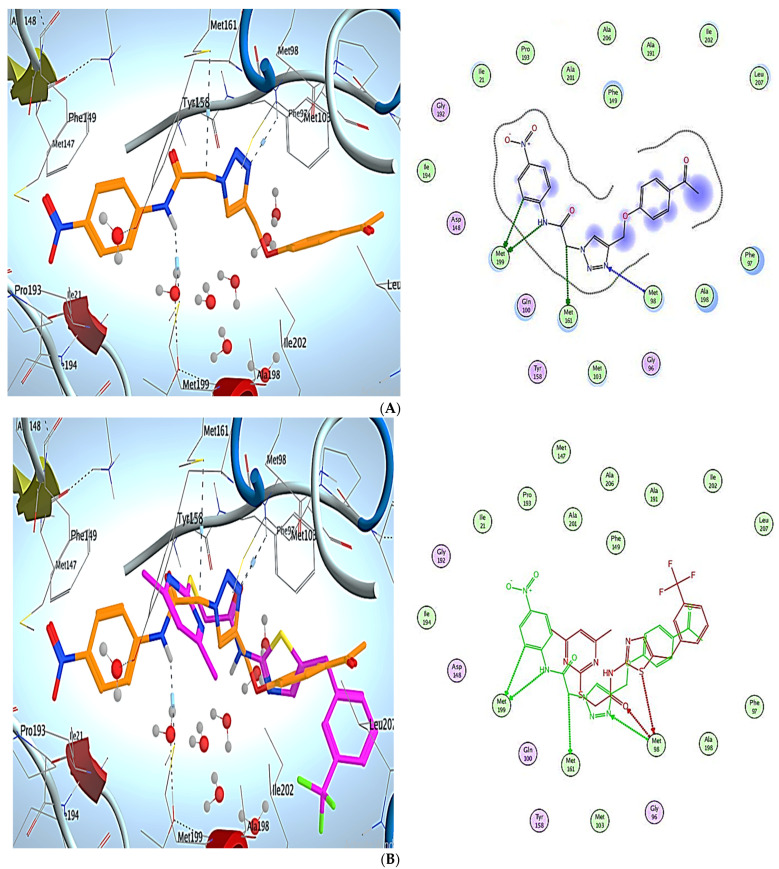
(**A**): Docking and binding pattern of compound **10** into InhA active site (PDB ID: 4UVD) in 2D (**right panel**) and 3D (**left panel**). (**B**): An overlay of the docked pose of compound **10** (brown) with the co-crystallized ligand (purple) into InhA active site in 2D (**right panel**) and 3D (**left panel**).

**Figure 7:**
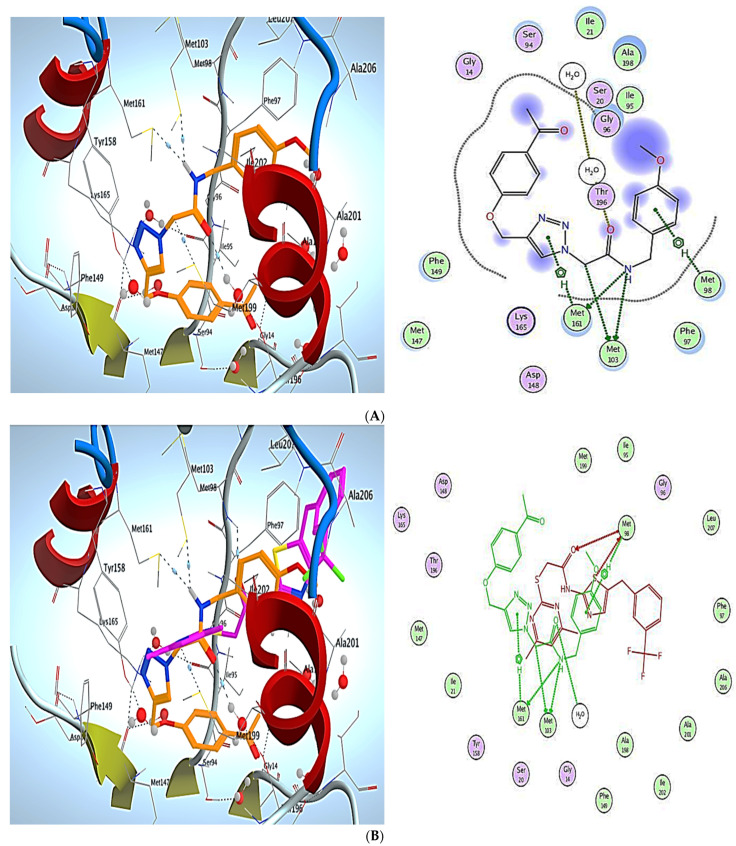
(**A**) Docking and binding pattern of compound **14** into InhA active site (PDB ID: 4UVD) in 2D (**right panel**) and 3D (**left panel**). (**B**): An overlay of the docked pose of compound **14** (brown) with the co-crystallized ligand (purple) into InhA active site in 2D (**right panel**) and 3D (**left panel**).

**Figure 8:**
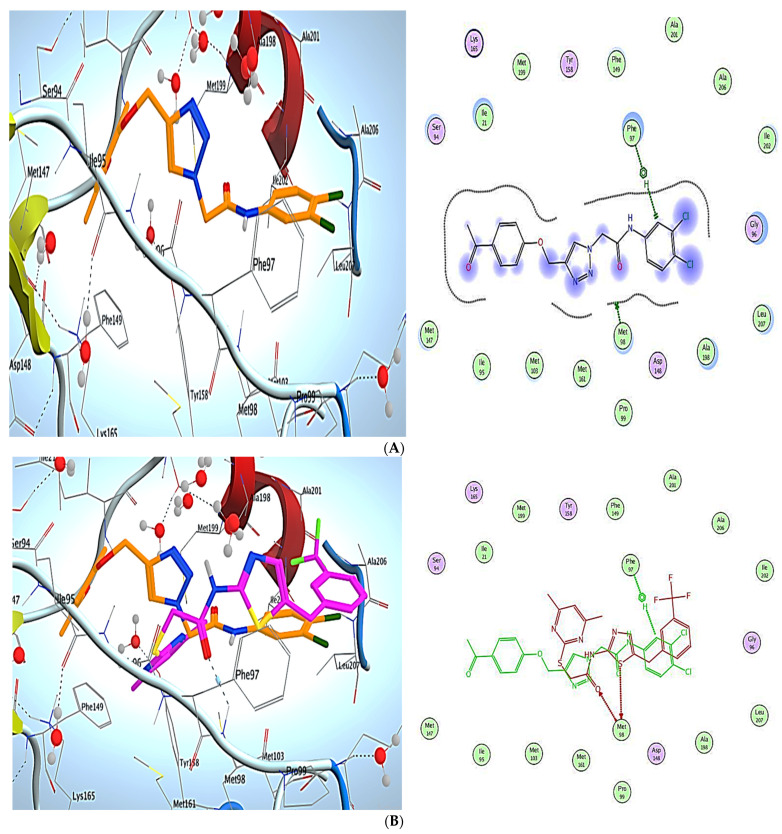
(**A**): Docking and binding pattern of compound **11** into InhA active site (PDB ID: 4UVD) in 2D (**right panel**) and 3D (**left panel**). (**B**): An overlay of the docked pose of compound **11** (brown) with the co-crystallized ligand (purple) into InhA active site in 2D (**right panel**) and 3D (**left panel**).

**Figure 9:**
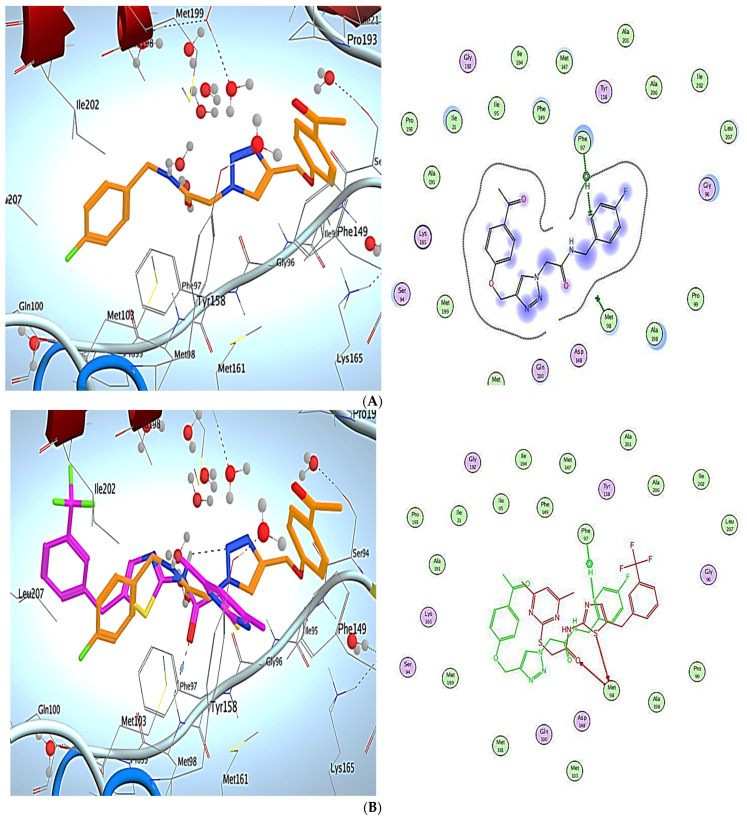
(**A**): Docking and binding pattern of compound **12** into InhA active site (PDB ID: 4UVD) in 2D (**right panel**) and 3D (**left panel**). (**B**): An overlay of the docked pose of compound **12** (brown) with the co-crystallized ligand (purple) into InhA active site in 2D (**right panel**) and 3D (**left panel**).

**Figure 10:**
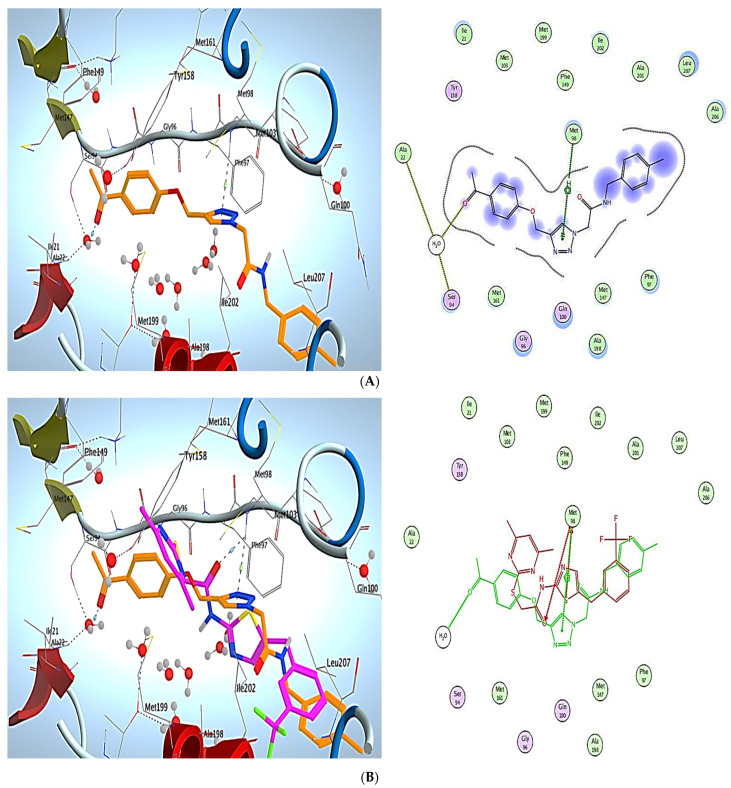
(**A**): Docking and binding pattern of compound **13** into InhA active site (PDB ID: 4UVD) in 2D (**right panel**) and 3D (**left panel**). (**B**): An overlay of the docked pose of compound **13** (brown) with the co-crystallized ligand (purple) into InhA active site in 2D (**right panel**) and 3D (**left panel**).

**Figure 11:**
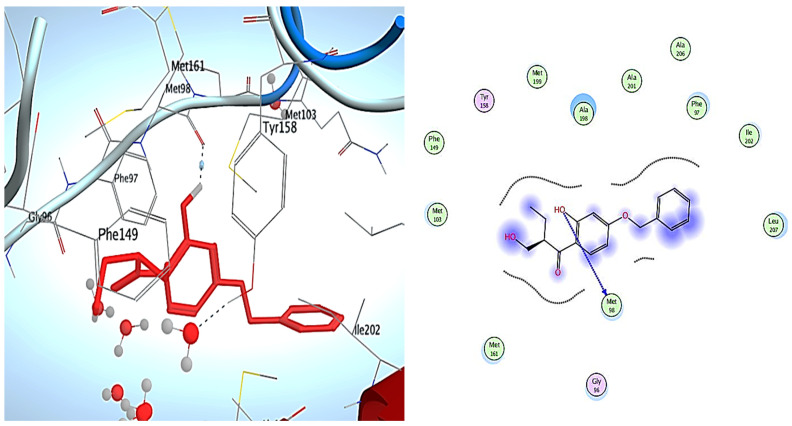
Docking and binding pattern of compound Lead structure **B** into InhA active site (PDB ID: 4UVD) in 2D (**right panel**) and 3D (**left panel**).

The most active compounds, 9, 10, and 14, were found to be at the best-docked position in the active site of the inhA enzyme (4UVD), with binding energy scores (S) of −8.63, −8.97, and −9. kcal/mol and RMSDs of 1.78, 1.85, and 1.91, respectively. With key amino acids inside the pocket, these compounds can create a non-covalent bonding interaction. For instance, the compound 9 triazole ring forms a hydrophobic bond of 3.86 Å with Met98. Similarly, the compound 10 triazole ring forms a hydrogen bond of 3.25 Å with Met98. Along the same track, the compound 14 4-methoxy benzyl ring can interact via a hydrophobic bond of 4.59 Å with met98. Moreover, Met103 forms hydrogen bonds of 4.15 Å and 3.83 Å with compound **9** triazole ring and acetamide nitrogen of compound 14, respectively. Furthermore, Met161 shares hydrogen bonds of 4.35 and 3.88 Å with acetamide linker of compounds **10** and **14**, respectively. Figure 5A,B, Figure 6A,B and Figure 7A,B. Accordingly, the importance of triazole scaffold and acetamide linker in future design of InhA inhibitors cannot be neglected. Compounds 11,12, and 13, on the other hand, which exhibit less invitro activity, showed main interactions with Met98. For example, the acetamide oxygen of compounds 11 and 12 forms hydrogen bonds of 3.12 Å, whereas compound 13, triazole ring, forms a hydrophobic interaction of 4.09 Å with Met98. There were no interactions between compounds 11, 12, and 13 and Met 103 and Met 161, which may explain why these compounds had lower in vitro activity (Figure 7A,B, Figure 8A,B, Figure 9A,B and Figure 10A,B). The reported InhA-Inhibitors Lead structure B IC_50_ = 0.38 μM was selected to dock into the 4UVD active site. Lead structure B showed (S) −7.39 kcal/mol and (RMSD) of 1.05 can occupy the active site of the enzyme through a hydrogen bond of 3.04 Å between 2-hydroxygroup and Met98 (Figure 11).

## 4. Experimental Methods

### 4.1. Chemistry

#### 4.1.1. General Information

All reagents and solvents used were of the highest quality of analytical reagent grade and were used without further purification. Melting points were measured on a Stuart Scientific SMP1 and are uncorrected. TLC was performed on UV fluorescent Silica gel Merck 60 F254 plates, and the spots were visualized using a UV lamp (254 nm). SHIMADZU FTIR-Affinity-1S spectrometer was used for identification of functional groups in the range of 400–4000 cm^−1^. The NMR spectra were run with Bruker spectrometers (500 and 400 MHz) in the presence of TMS as internal reference. All azides used in this study were prepared according to reported procedures [37,38,39,40]

#### 4.1.2. Synthesis Andof 1-(4-(Prop-2-Yn-1-yloxy) phenyl) ethan-1-one (**2**)

A mixture of 4-hydroxyacetophenone (**1**) (10 mmol) in dimethylformamide (10 mL) and potassium carbonate (12 mmol) was stirred for 1 h. Then, propargyl bromide (1.2 mmol) was added, and the stirring was continued for 4 h at 80 °C until the consumption of the starting material as indicated by TLC (hexane-ethyl acetate). After cooling, the mixture was poured onto crushed ice water and the precipitate formed was filtered, washed with water, dried and crystallized from ethanol to give the targeted *O*-propargylated acetophenone **2** as pale yellow powder in 91% yield, mp: 150–151 °C IR (*υ*, cm^−1^): 1250 (C-O), 1600 (C=C), 1680 (C=O), 2100 (C≡C), 2900 (CH-Al), 3070 (CH-Ar), 3210 (≡CH). ^1^H NMR (400 MHz, CDCl_3_) δ_H_ = 2.57 (bs, 4H, CH_3_ and ≡CH), 4.77 (d, 2H, *J* = 4.0 Hz, OCH_2_), 7.03 (d, 2H, *J* = 4.0 Hz, Ar-H), 7.97 (d, 2H, *J* = 12.0 Hz, Ar-H). ^13^C NMR (100 MHz, CDCl_3_): δ_C_= 21.70 (CH_3_); 51.08 (OCH_2_); 71.48 (C≡CH); 73.00 (C≡CH); 109.68, 109.80, 125.21, 125.50, 125.80, 125.95, 126.23, 156.51, 156.51 (Ar-C); 192.23 (C=O) Figures S1–S3.

##### 2-(4-((4-Acetylphenoxy)methyl)-1H-1,2,3-triazol-1-Yl)-N-(2-fluorophenyl)acetamide (**9**)

This compound was obtained as pale yellow solid in 87% yield, mp: 179–180 °C. IR (*υ*, cm^−1^): 1550 (C=C), 1670 (C=O), 1705 (C=O), 3060 (CH-Ar), 3300 (NH). ^1^H NMR (500 MHz, DMSO-*d*_6_): δ_H_ = 2.48 (s, 3H, CH_3_ overlapped with DMSO-*d*_6_), 5.24 (s, 2H, OCH_2_), 5.41 (s, 2H, NCH_2_), 7.13–7.25 (m, 5H, Ar-H), 7.86–7.89 (m, 1H, Ar-H), 7.91 (d, 2H, *J* =10 Hz, Ar-H), 8.25 (s, 1H, CH-1,2,3-triazole), 10.29 (s, 1H, NH). ^13^C NMR (125 MHz, DMSO-*d*_6_): δ_C_ = 27.16 (CH_3_); 52.96 (OCH_2_); 61.74 (NCH_2_); 115.34, 115.89, 116.40, 124.16, 124.97, 125.96, 126.08, 126.19, 126.32, 127.12, 130.84, 142.49, 162.40, 165.23 (Ar-C, CONH); 196.89 (C=O). ^19^F NMR (377 MHz, DMSO-*d*_6_): δ_F_ = −124.75 to −124.69 (m, 1F, Ar-F) Figures S4–S7.

##### 2-(4-((4-Acetylphenoxy)methyl)-1H-1,2,3-triazol-1-Yl)-N-(4-nitrophenyl)acetamide (**10**)

This compound was obtained as white solid in 89% yield, mp: 185–186 °C. IR (*υ*, cm^−1^): 1580 (C=C), 1680 (C=O), 1710 (C=O), 3030 (Ar CH), 3325 (NH). ^1^H NMR (400 MHz, DMSO-*d*_6_): δ_H_ = 2.48 (s, 3H, CH_3_ overlapped with DMSO-*d*_6_), 5.19 (s, 2H, OCH_2_), 5.74 (s, 2H, NCH_2_), 7.01–7.08 (m, 2H, Ar-H), 7.18–7.28 (m, 4H, Ar-H), 7.73–7.77 (m, 2H, Ar-H), 8.38 (s, 1H, CH-1,2,3-triazole), 9.82 (s, 1H, NH). ^13^C NMR (100 MHz, DMSO-*d*_6_): δ_C_ = 27.31 (CH_3_); 52.37 (OCH_2_); 61.95 (NCH_2_); 115.21, 115.71, 129.16, 129.74, 130.26, 132.38, 133.49, 133.83, 148.91, 158.63, 161.96, 163.33 (Ar-C, CONH); 192.06 (C=O). 

##### 2-(4-((4-Acetylphenoxy)methyl)-1H-1,2,3-triazol-1-Yl)-N-(3,4-dichlorophenyl)acetamide (**11**)

This compound was obtained as pale yellow solid in 89% yield, mp: 205–206 °C. IR (*υ*, cm^−1^): 1540 (C=C), 1670 (C=O), 1705 (C=O), 3090 (CH-Ar), 3325 (NH). ^1^H NMR (400 MHz, DMSO-*d*_6_): δ_H_ = 2.54 (s, 3H, CH_3_ overlapped with DMSO-*d*_6_), 5.28 (s, 2H, OCH_2_), 5.38 (s, 2H, NCH_2_), 7.17 (d, 2H, *J* = 8.0 Hz, Ar-H), 7.45–7.60 (m, 2H, Ar-H), 7.93–7.95 (m, 3H, Ar-H), 8.30 (s, 1H, CH-1,2,3-triazole), 10.85 (s, 1H, NH). ^13^C NMR (100 MHz, DMSO-*d*_6_): δ_C_ = 26.88 (CH_3_); 52.62 (OCH_2_); 61.70 (NCH_2_); 114.98, 119.79, 120.96, 125.89, 127.21, 130.52, 130.90, 130.98, 131, 17, 131.36, 131.63, 138.80, 142.65, 162.30, 165.25 (Ar-C, CONH); 197.09 (C=O) Figures S8–S10.

##### 2-(4-((4-Acetylphenoxy)methyl)-1H-1,2,3-triazol-1-Yl)-N-(4-fluorobenzyl)acetamide (**12**)

This compound was obtained as pale yellow solid in 88% yield, mp: 165–166 °C. IR (*υ*, cm^−1^): 1540 (C=C), 1670 (C=O), 1695 (C=O), 3070 (CH-Ar), 3290 (NH). ^1^H NMR (400 MHz, DMSO-*d*_6_): δ_H_ = 2.51 (s, 3H, CH_3_ overlapped with DMSO-*d*_6_), 4.30 (d, 2H, *J* = 8.0 Hz, NHCH_2_), 5.19 (s, 2H, OCH_2_), 5.25 (s, 2H, NCH_2_), 7.15–7.16 (m, 4H, Ar-H), 7.30–7.33 (m, 2H, Ar-H), 7.94 (d, 2H, *J* = 8.0 Hz, Ar-H), 8.25 (s, 1H, CH-1,2,3-triazole), 8.89 (s, 1H, NH). ^13^C NMR (100 MHz, DMSO-*d*_6_): δ_C_ = 26.88 (CH_3_); 42.35 (NHCH_2_); 52.05 (OCH_2_); 61.71 (NCH_2_); 114.97, 115.46, 115.67, 127.02, 129.74, 129.80, 130.51, 130.97, 135.31, 160.48, 162.31, 162.93, 165.93 (Ar-C, CONH); 197.07 (C=O). ^19^F NMR (172 MHz, DMSO-*d*_6_): δ_F_ = −118.56 to −118.48 (m, 1F, Ar-F) Figures S11–S14.

##### 2-(4-((4-Acetylphenoxy)methyl)-1H-1,2,3-triazol-1-Yl)-N-(4-methylbenzyl)acetamide (**13**)

This compound was obtained as pale yellow solid in 89% yield, mp: 180–182 °C. IR (*υ*, cm^−1^): 1575 (C=C), 1670 (C=O), 1695 (C=O), 3040 (CH-Ar), 3295 (NH). ^1^H NMR (500 MHz, DMSO-*d*_6_): δ_H_ = 2.23 (s, 3H, CH_3_), 2.43 (s, 3H, COCH_3_ overlapped with DMSO-*d*_6_),4.24 (d, 2H, *J* = 5 Hz, NHCH_2_), 5.14 (s, 2H, OCH_2_), 5.23 (s, 2H, NCH_2_), 7.09–7.13 (m, 6H, Ar-H), 7.91 (d, 2H, *J* = 5 Hz, Ar-H), 8.20 (s, 1H, CH-1,2,3-triazole), 8.77 (t, 1H, *J* = 5 Hz, NH). ^13^C NMR (125 MHz, DMSO-*d*_6_): δ_C_ = 21.19 (CH_3_), 26.96 (COCH_3_); 40.38 (NHCH_2_); 52.15 (OCH_2_); 61.78 (NCH_2_); 115.05, 126.94, 127.93, 129.43, 129.45, 130.63, 131.00, 136.17, 136.64, 142.44, 162.41, 165.82 (Ar-C, CONH); 196.85 (C=O) Figures S15–S17.

##### 2-(4-((4-Acetylphenoxy)methyl)-1H-1,2,3-triazol-1-Yl)-N-(4-methoxybenzyl)acetamide (**14**)

This compound was obtained as pale yellow solid in 90% yield, mp: 125–126 °C. IR (*υ*, cm^−1^): 1580 (C=C), 1680 (C=O), 1700 (C=O), 3070 (CH-Ar), 3350 (NH). ^1^H NMR (400 MHz, DMSO-*d*_6_): δ_H_ = 2.46 (s, 1H, CH_3_ overlapped with DMSO-*d*_6_), 3.69 (s, 3H, OCH_3_), 4.21 (d, 2H, *J* = 4.0 Hz, NHCH_2_), 5.13 (s, 2H, OCH_2_), 5.23 (s, 2H, NCH_2_), 6.86 (d, 2H, *J* = 4.0 Hz, Ar-H), 7.13 (d, 2H, *J* = 8.0 Hz, Ar-H), 7.18 (d, 2H, *J* = 8.0 Hz, Ar-H), 7.91 (d, 2H, *J* = 8.0 Hz, Ar-H), 8.20 (s, 1H, CH-1,2,3-triazole), 8.75 (t, 1H, *J*= 4.0 Hz, NH). ^13^C NMR (100 MHz, DMSO-*d*_6_): δ_C_ = 26.96 (COCH_3_); 42.39 (NHCH_2_); 52.15 (OCH_2_); 55.60 (OCH_3_); 61.78 (NCH_2_); 114.29, 115.05, 129.34, 130.63, 131.00, 131.13, 158.91, 162.41, 165.74 (Ar-C, CONH); 196.86 (C=O) Figures S18–S20.

### 4.2. Enzymatic Inhibition Experiments

*M. tuberculosis* InhA was overexpressed in *E. coli* while NADH was obtained from Sigma-Aldrich. The concentration of the pool INH–NAD was determined on the basis of ε_330_ equaled 6900 M^−1^ cm^−1^. The substrate 2-*trans*-decenoyl-CoA concentration was determined on the basis of ε_260_ equaled 22,600 M^−1^ cm^−1^.

For the inhibition assays with InhA, the pre-incubation reactions were performed in 80 μL (total volume) of 30 mM PIPES buffer solution, 150 mM NaCl, pH 6.8 at 25 °C containing 70 nM InhA and the tested compounds (at different concentrations). DMSO was used as co-solvent and its final concentration was 0.5%. After 2 h of pre-incubation, the addition of 35 μM substrate (*trans*-2-decenoyl-CoA) and 200 μM cofactor (NADH) initiated the reaction which was measured at 25 °C and at 340 nm (oxidation of NADH) using a spectrophotometer (PG-T80, PG Instruments lmited woodway lane, Leicester, UK). Control reactions were done under the same conditions but without the ligands. The pool of INH–NAD adducts was used as a positive control. The initial rates of the reactions were calculated. Rifampicin was used as a reference commercially known drug. The inhibition percentage of each compound was measured at 10 µM and the compounds’ inhibitory activity was expressed as the IC_50_ inhibition of InhA activity with respect to the control experiments [41].

### 4.3. Docking Study

Computer-aided docking experiments were performed using Molecular Operating Environment (MOE 2014.0802) software (Chemical Computing Group, Montreal, QC, Canada).

#### 4.3.1. Preparation of the Protein Crystal Structures

The protein data bank provided the X-ray crystal structures of InhA (PDB ID: 4UVD) with its co-crystallized ligand (HRW). Explicit hydrogen atoms were added to the receptor complex structure and partial charges were calculated. The preparation was completed with structure preparation module employing protonated 3D function.

The co-crystal ligand (HRW) was extracted from their corresponding proteins and used as reference molecules for the validation study.

#### 4.3.2. Preparation of the Selected Compounds for Docking

The target compounds were constructed using the builder module of MOE. The compounds were then collected in a database and prepared by adding hydrogens, calculating partial charges and energy minimizing using Force field MMFF94x.

The top-scored conformation with the best binding interactions detected by the MOE search algorithm and scoring function was the basis for the selection of the docking poses. In addition, binding energy scores, formation of binding interaction with the neighboring amino acid residues, and the relative positioning of the docked poses in comparison to the co-crystallized ligands were the factors determining the binding affinities to the binding pockets of the enzyme.

## 5. Conclusions

To target M. tuberculosis’ enoyl-acyl carrier protein reductase, the CuAAC of 1-(4-(prop-2-yn-1-yloxy) phenyl) ethanone with suitable acetamide azides was employed to develop and synthesize the 1,2,3-triazole molecular series. At a dose of 10 µM, all of the synthetic inhibitors completely inhibited the InhA enzyme and outperformed rifampicin in terms of activity. The most promising InhA inhibitors were 9,10, and 14, which had IC_50_ values of 0.005, 0.008, and 0.002 µM, respectively. As a result, our compounds may open the path for new, extremely effective anti-TB drugs.

## Figures and Tables

**Table 1:** InhA inhibition (IC_50_) of **9-14** with respect to rifampicin.

Code	Structure	IC_50_ (µM)
**Rifampicin**	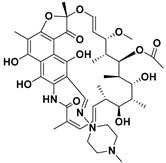	**8.50**
**Isoniazide**	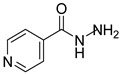	**0.054**
**9**	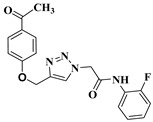	**0.005**
**10**	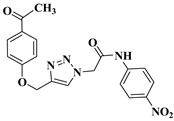	**0.008**
**11**	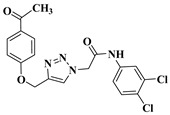	**0.043**
**12**	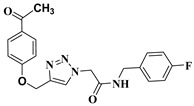	**0.041**
**13**	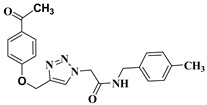	**0.084**
**14**	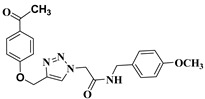	**0.002**

## Data Availability

Data is contained within the article and supplementary files.

## Supplementary Materials

The following are available online at https://www.mdpi.com/article/10.3390/ph15070799/s1, Figure S1: IR spectrum of compound **2**, Figure S2: ^1^H NMR spectrum of compound **2**, Figure S3: ^13^C NMR spectrum of compound **2**, Figure S4: IR spectrum of compound 9, Figure S5: 1H NMR spectrum of compound **9**, Figure S6: ^13^C NMR spectrum of compound **9**, Figure S7: ^19^F NMR spectrum of compound **9**, Figure S8: IR spectrum of compound **11**, Figure S9: ^1^H NMR spectrum of compound **11**, Figure S10: ^13^C NMR spectrum of compound **11**, Figure S11: IR spectrum of compound **12**, Figure S12: ^1^H NMR spectrum of compound **12**, Figure S13: ^13^C NMR spectrum of compound **12**, Figure S14: ^19^F NMR spectrum of compound **12**, Figure S15: IR spectrum of compound **13**, Figure S16: ^1^H NMR spectrum of compound **13**, Figure S17: ^13^C NMR spectrum of compound **13**, Figure S18: IR spectrum of compound **14**, Figure S19: ^1^H NMR spectrum of compound **14**, Figure S20: ^13^C NMR spectrum of compound **14**.
