# Supplementary material for: Design, Synthesis and Molecular Docking of Novel Acetophenone-1,2,3-Triazoles Containing Compounds as Potent Enoyl-Acyl Carrier Protein Reductase (InhA) Inhibitors
Source: Pharmaceuticals (Basel). 2022 Jun 27;15(7):799. doi: 10.3390/ph15070799 (PMC9316523; doi:10.3390/ph15070799)
Supplement: Supplementary file 1 [file pharmaceuticals-15-00799-s001.zip › pharmaceuticals-1711587-supplementary.pdf]

Supplementary Material

Design, Synthesis and Molecular Docking of Novel Acetophenone-1,2,3-triazoles containing compounds as potent Enoyl-Acyl Carrier Protein Reductase (InhA) inhibitors

FawziaFalehAlbelwi[1], Hanaa M. Abdu Mansour [1], Maram M. Elshatanofy[2], Yeldez El Kilany[2], Kamal Kandeel [3], Bassma H Elwakil[4], Mohamed Hagar,[2] Mohamed Reda Aouad [1],El Sayed H. El Ashry [2], Nadjet Rezki [1],* Maged A. El Sawy[5]

1. Department of Chemistry, Faculty of Science, Taibah University, Al-Madinah Al-Munawarah 30002, Saudi Arabia
2. Department of Chemistry, Faculty of Science, Alexandria University, Alexandria 21321, Egypt.
3. Department of Biochemistry, Faculty of Science, Alexandria University, MoharamBeik, 21547, Alexandria, Egypt, kamkandeel@yaho.com.
4. Department of Medical laboratory technology, Faculty of Applied Health Sciences Technology, Pharos University in Alexandria, Alexandria, Egypt. bassma.hassan@pua.edu.eg
5. Department of Pharmaceutical Chemistry, Faculty of Pharmacy. Pharos University, 21311, Alexandria, Egypt

* Correspondence: N.R. nadjetrezki@yahoo.fr

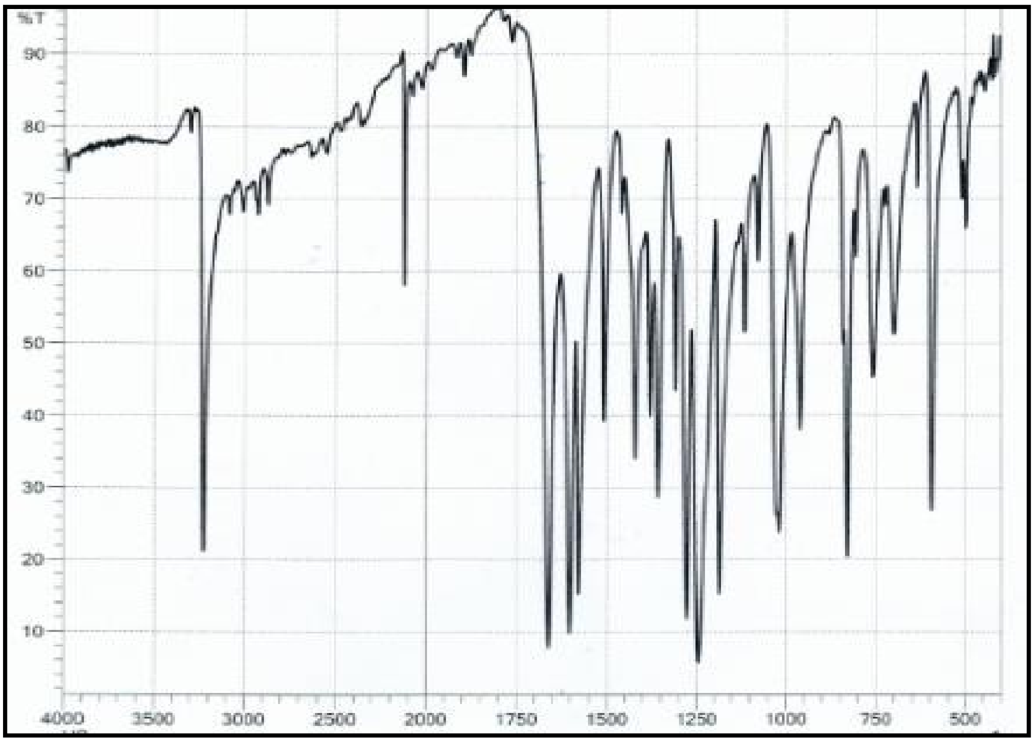

Figure S1: IR spectrum of compound **2**

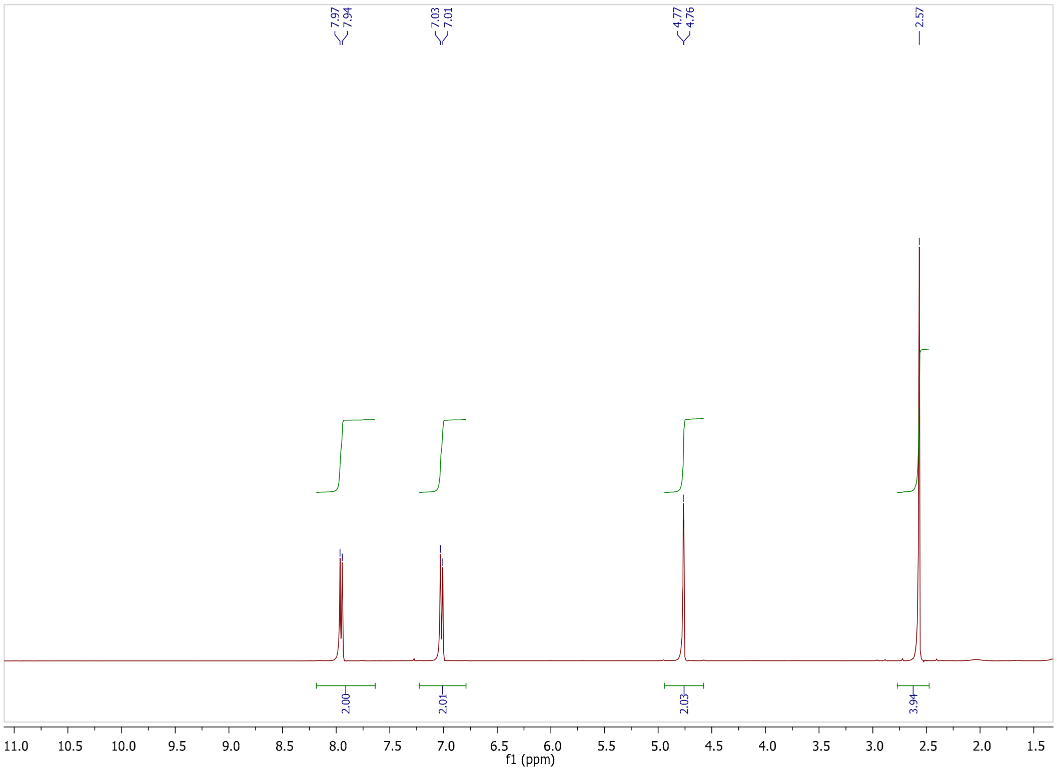

Figure S2: ^{1}H NMR spectrum of compound **2**

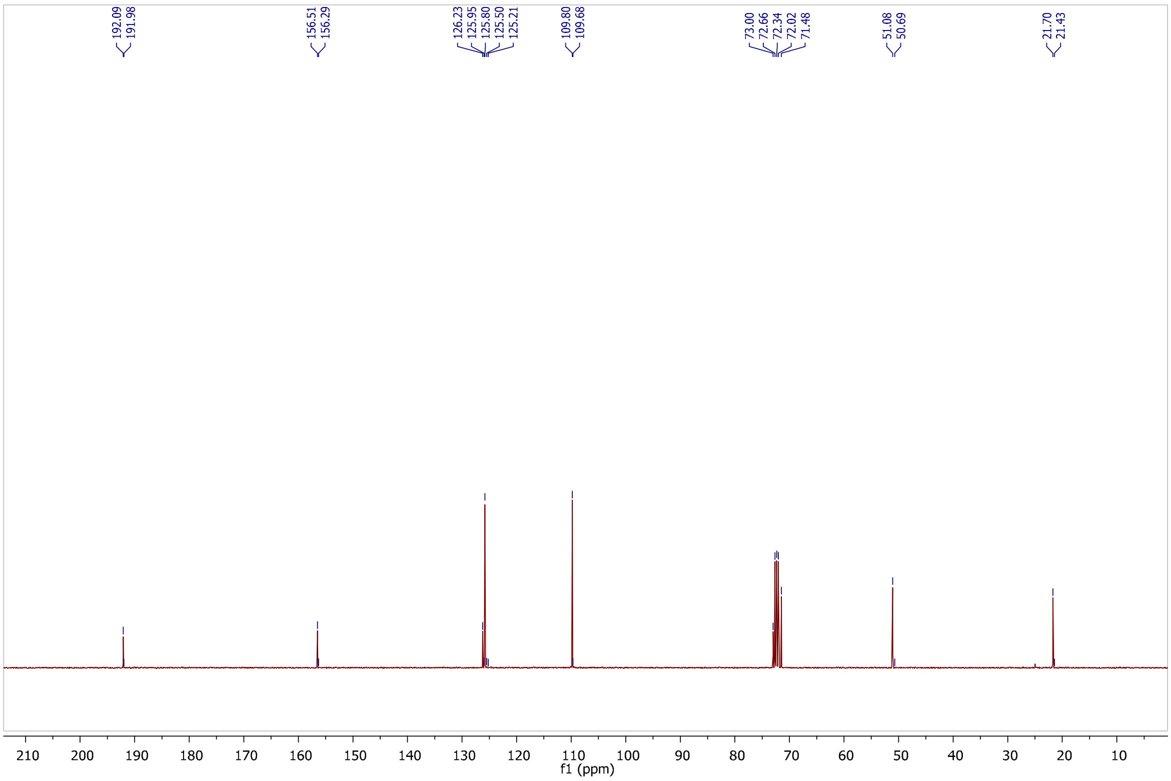

Figure S3: ^{13}C NMR spectrum of compound **2**

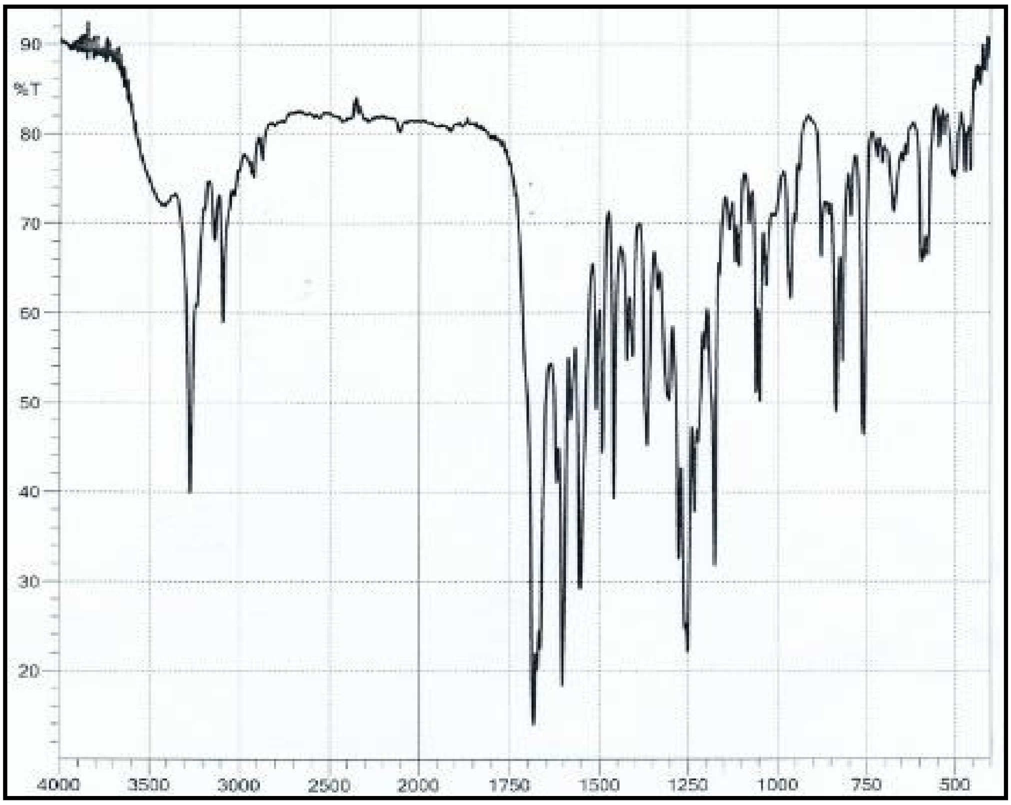

Figure S4: IR spectrum of compound **9**

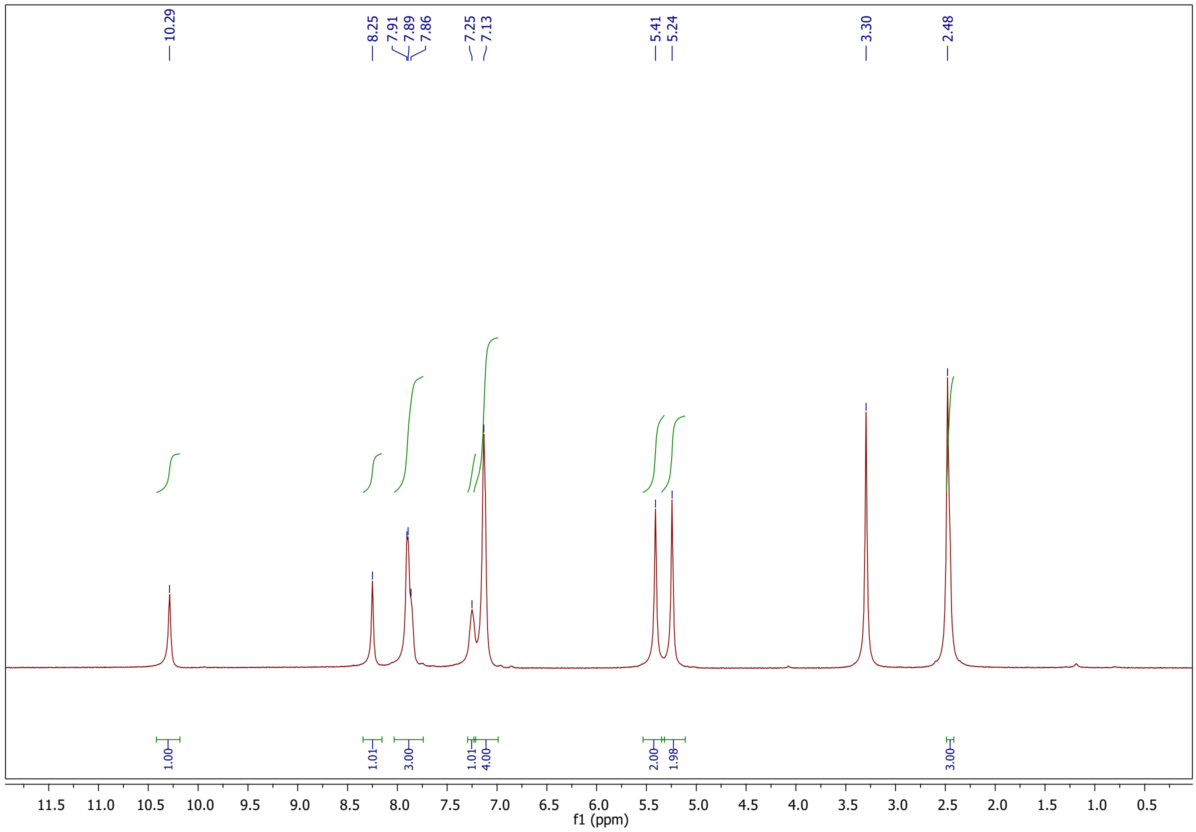

Figure S5: ^{1}H NMR spectrum of compound **9**

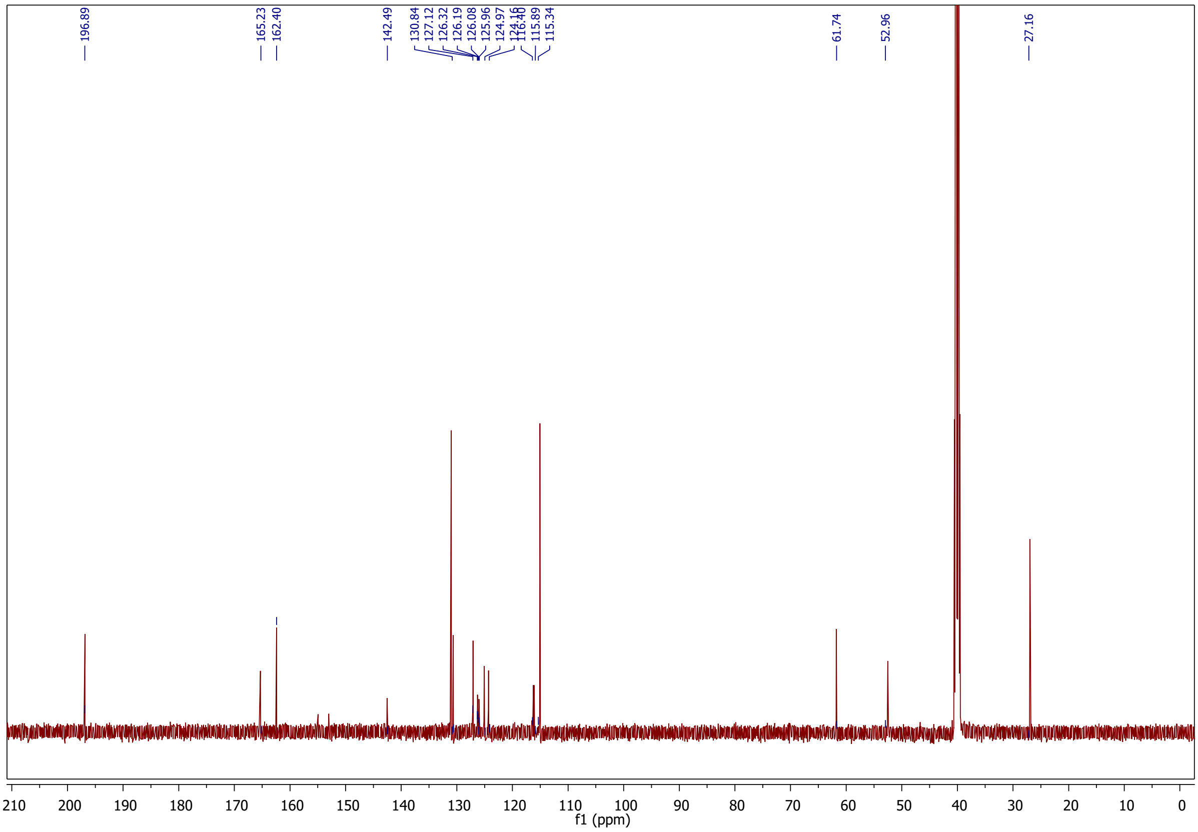

Figure S6: ^{13}C NMR spectrum of compound **9**

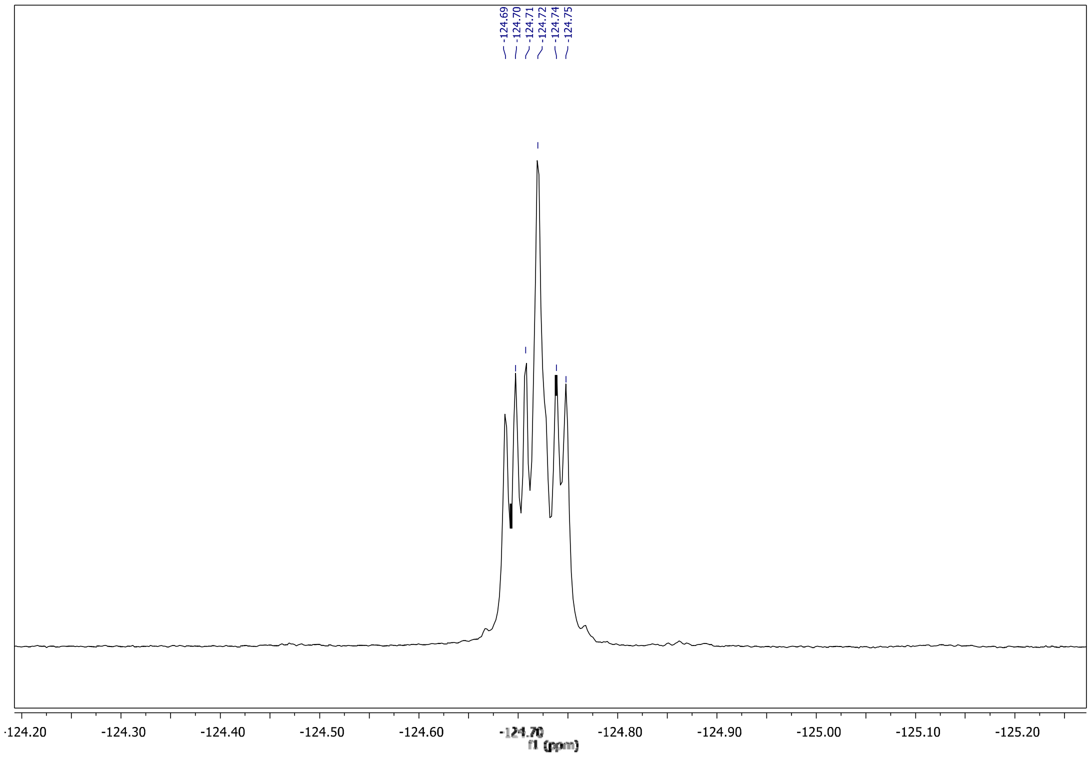

Figure S7: ^{19}F NMR spectrum of compound **9**

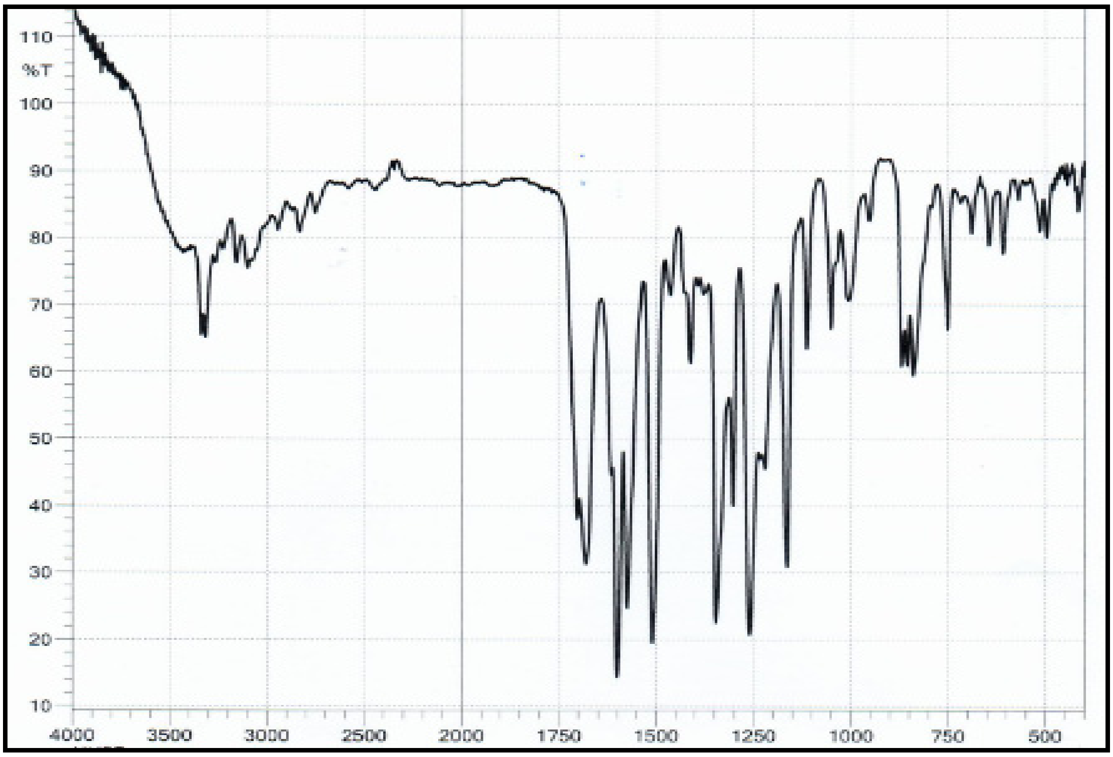

Figure S8: IR spectrum of compound **11**

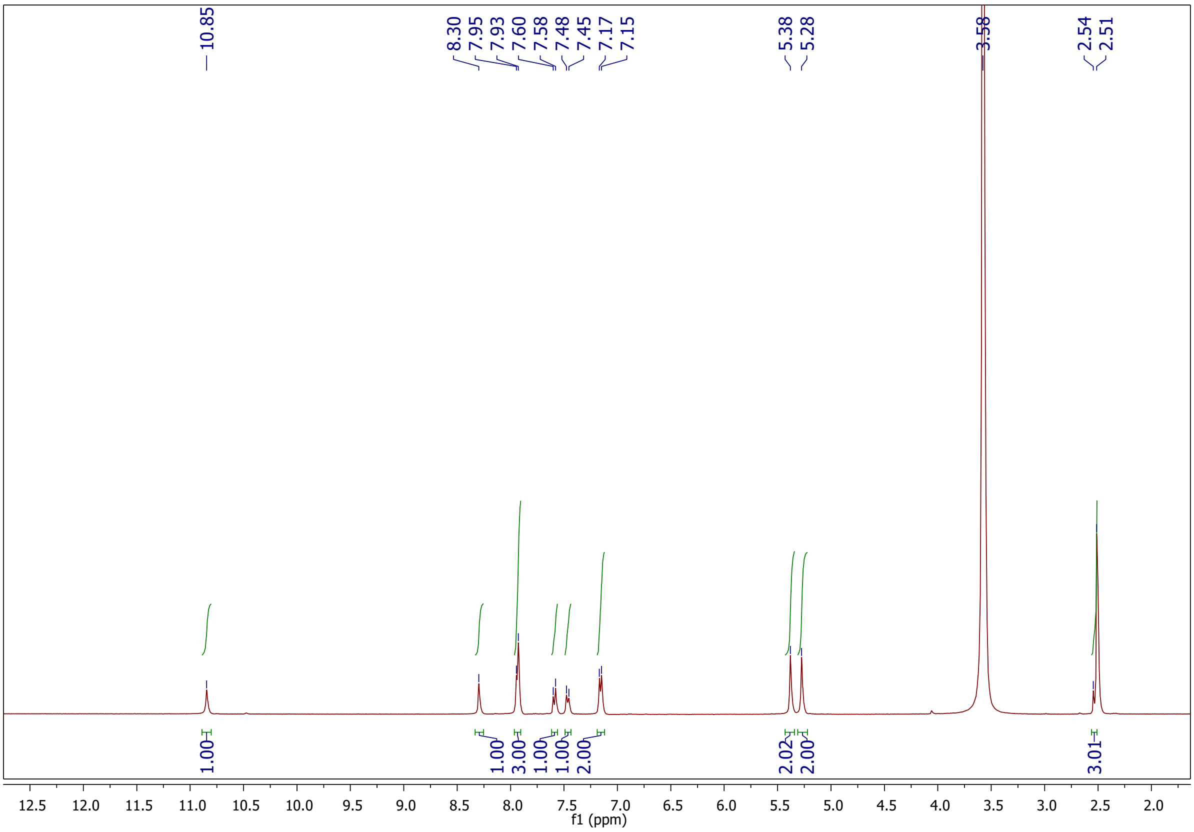

Figure S9: ^{1}H NMR spectrum of compound **11**

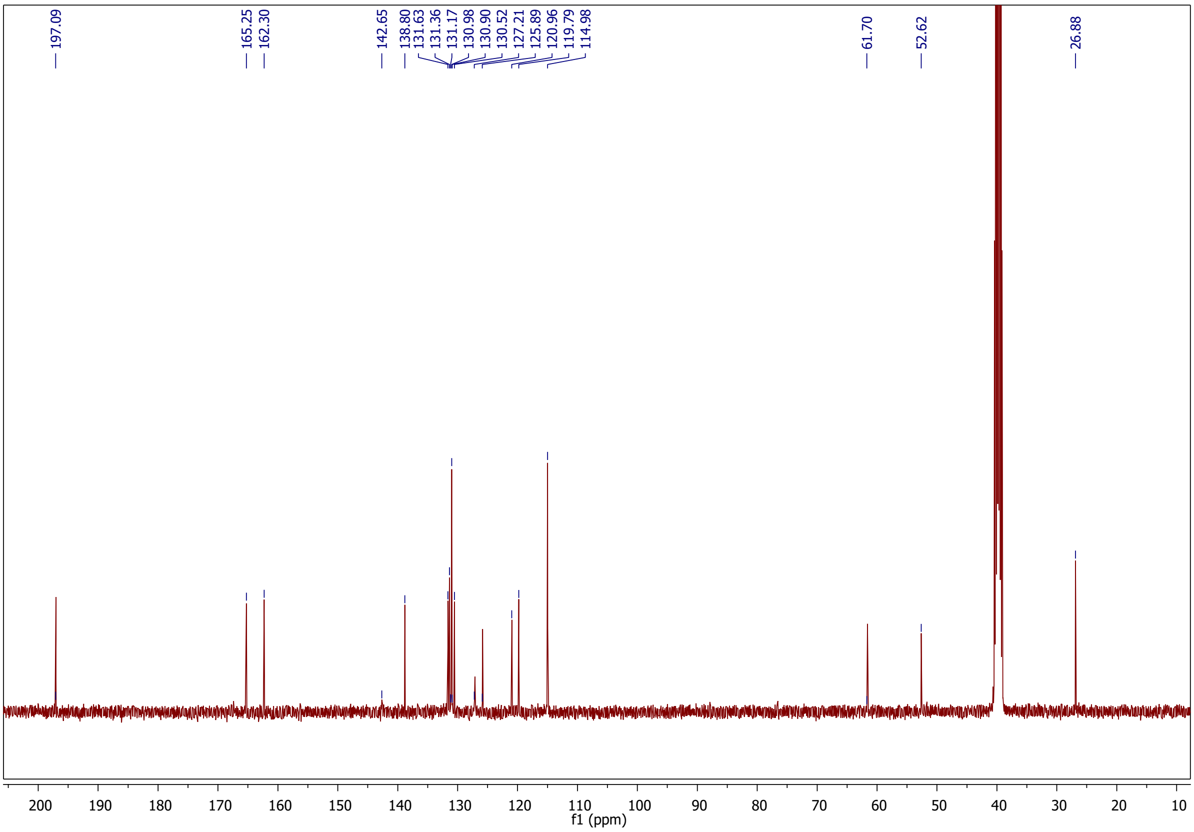

Figure S10: ^{13}C NMR spectrum of compound **11**

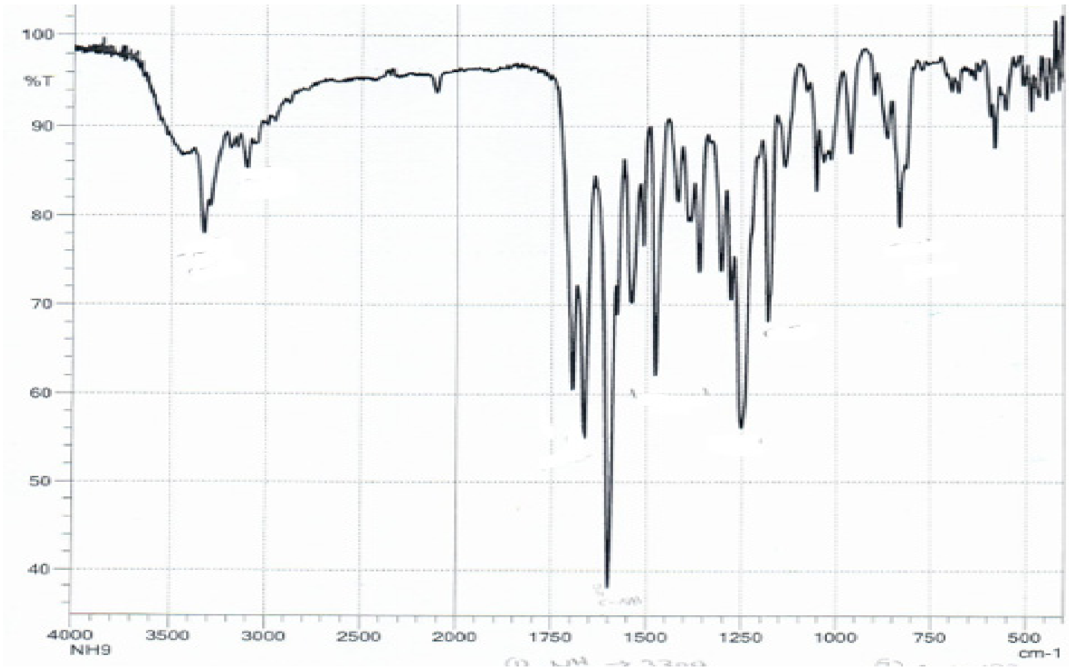

Figure S11: IR spectrum of compound **12**

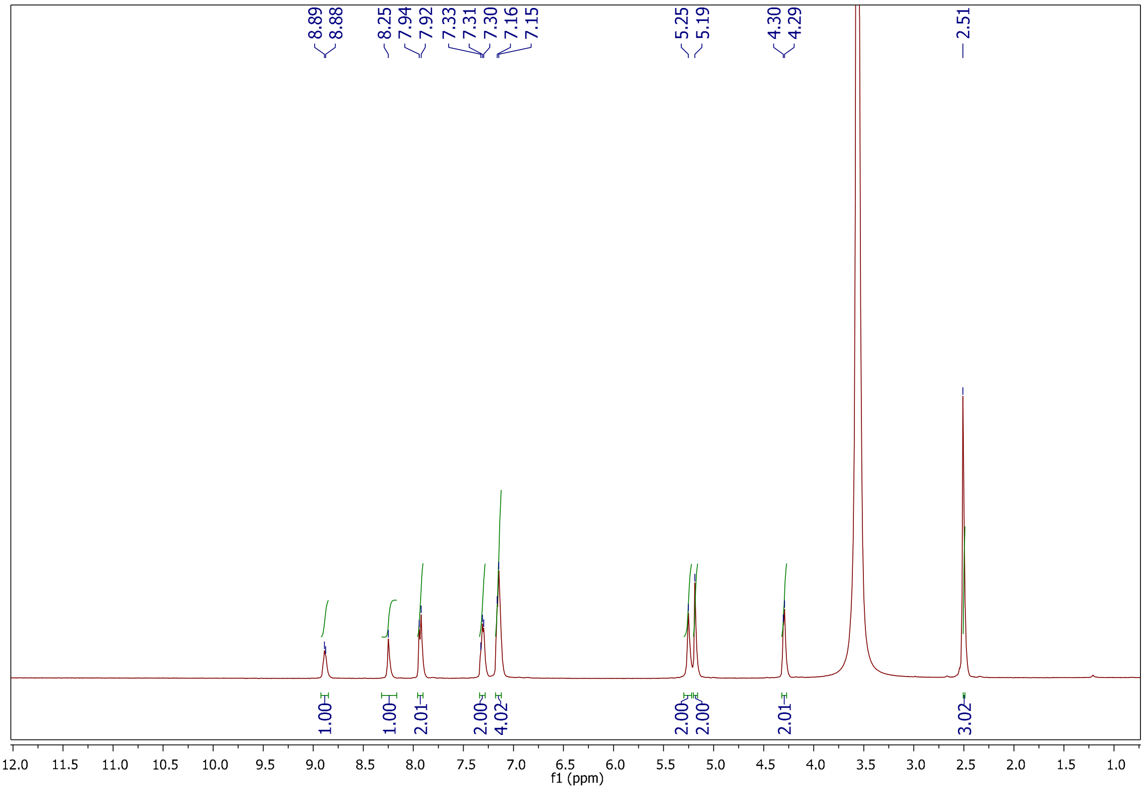

Figure S12: ^{1}H NMR spectrum of compound **12**

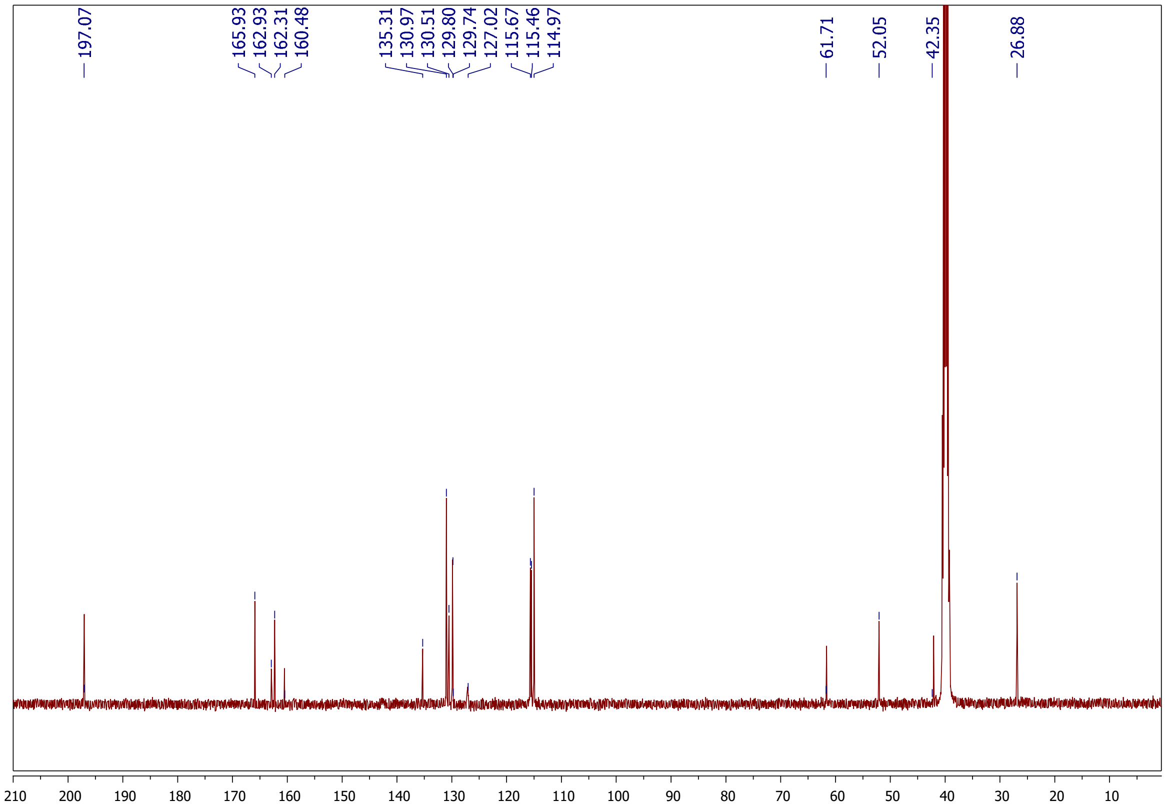

Figure S13: ^{13}C NMR spectrum of compound **12**

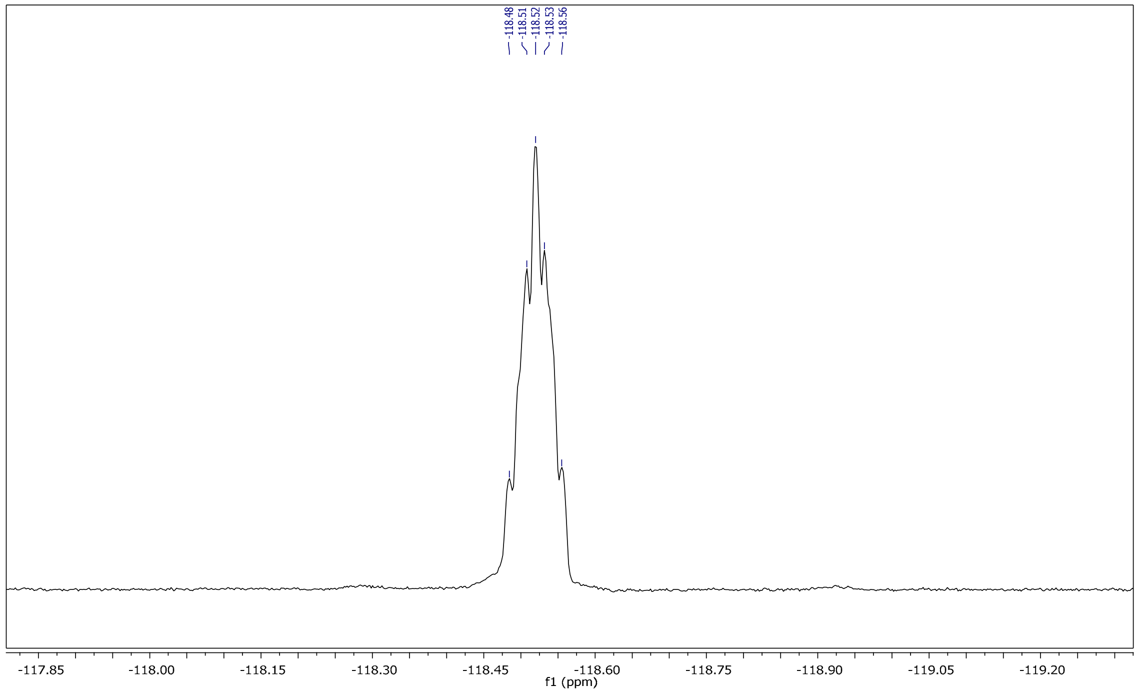

Figure S14: ^{19}F NMR spectrum of compound **12**

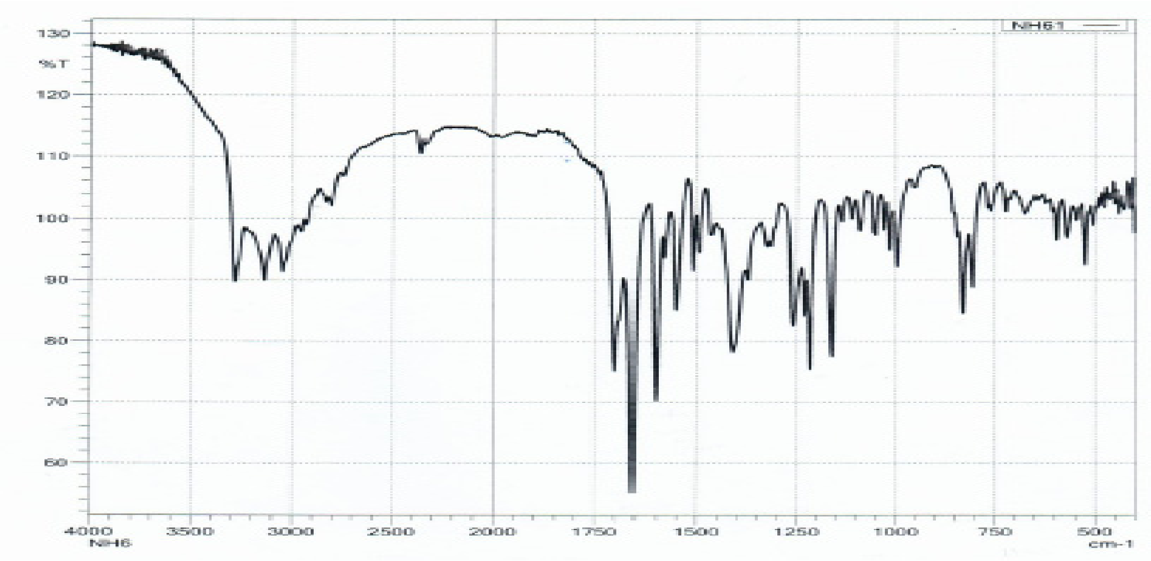

Figure S15: IR spectrum of compound **13**

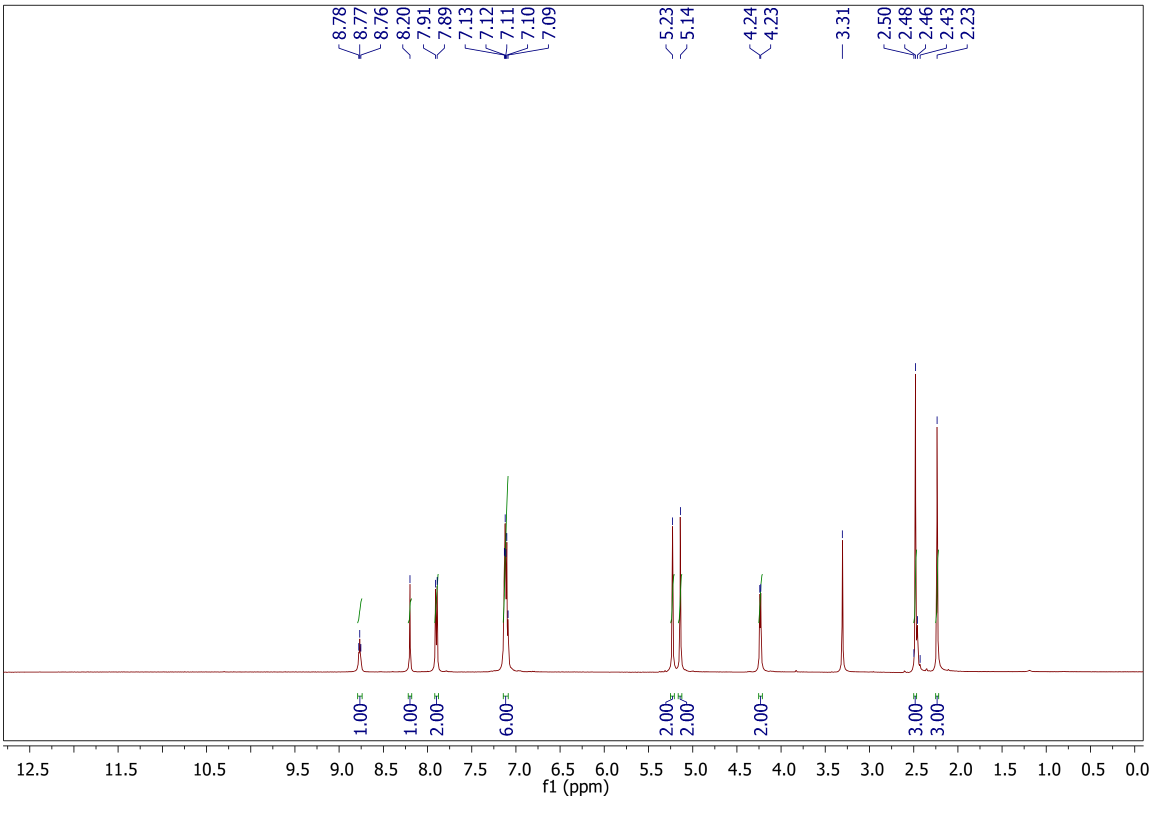

Figure S16: ^{1}H NMR spectrum of compound **13**

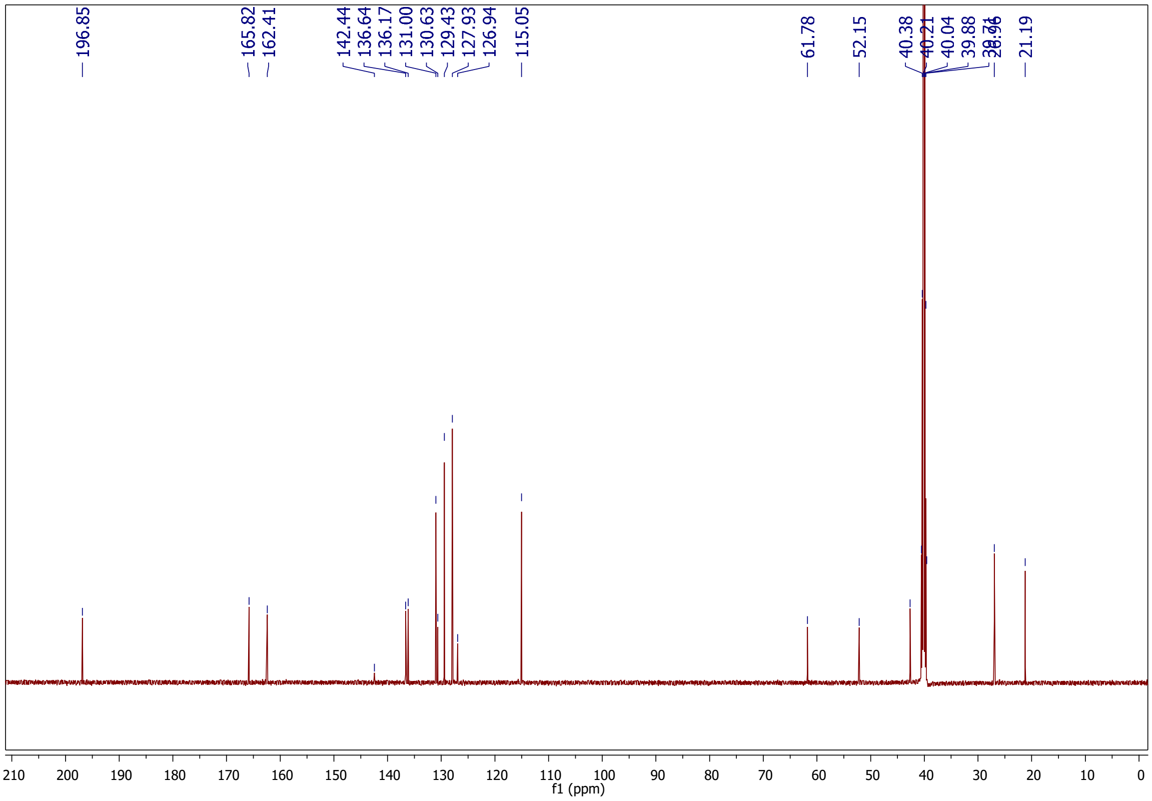

Figure S17: ^{13}C NMR spectrum of compound **13**

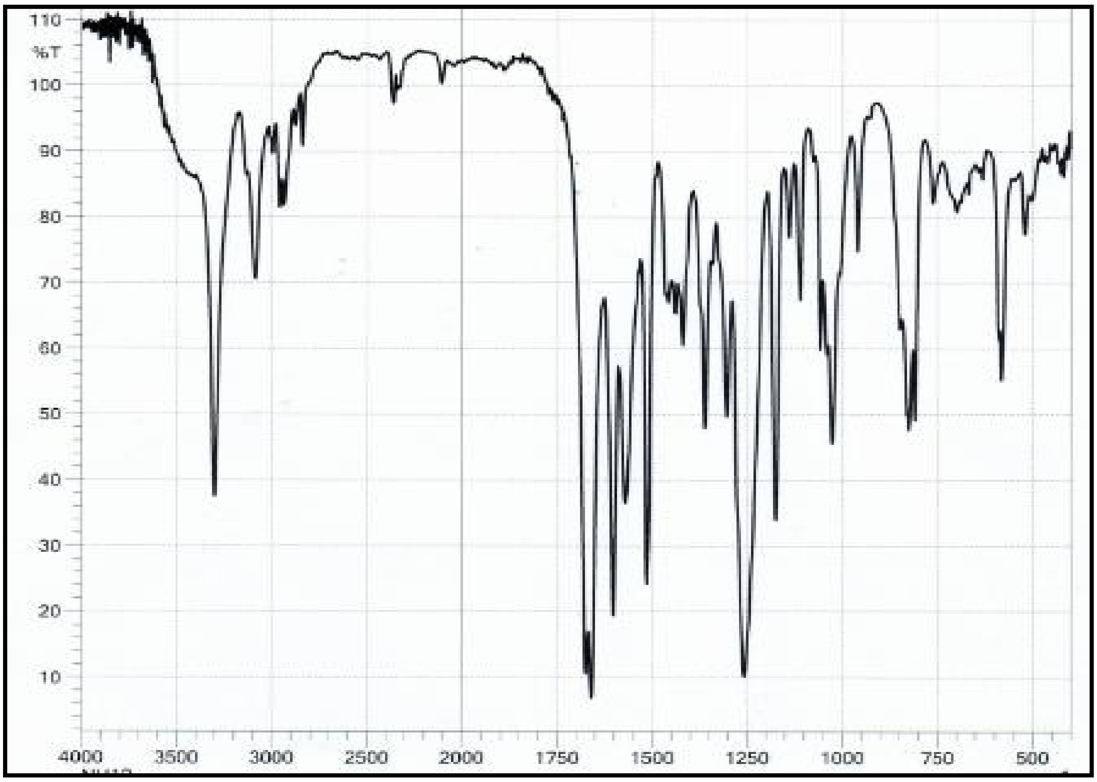

Figure S18: IR spectrum of compound **14**

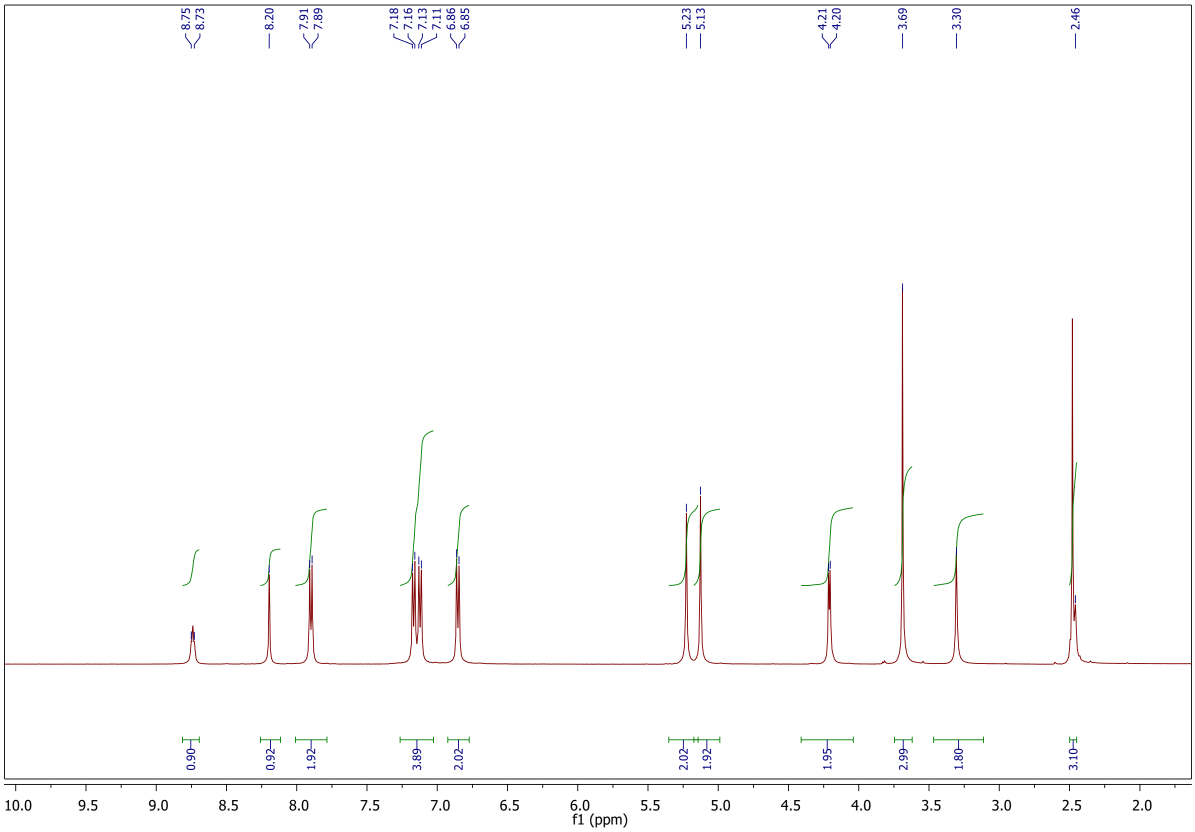

Figure S19: ^{1}H NMR spectrum of compound **14**

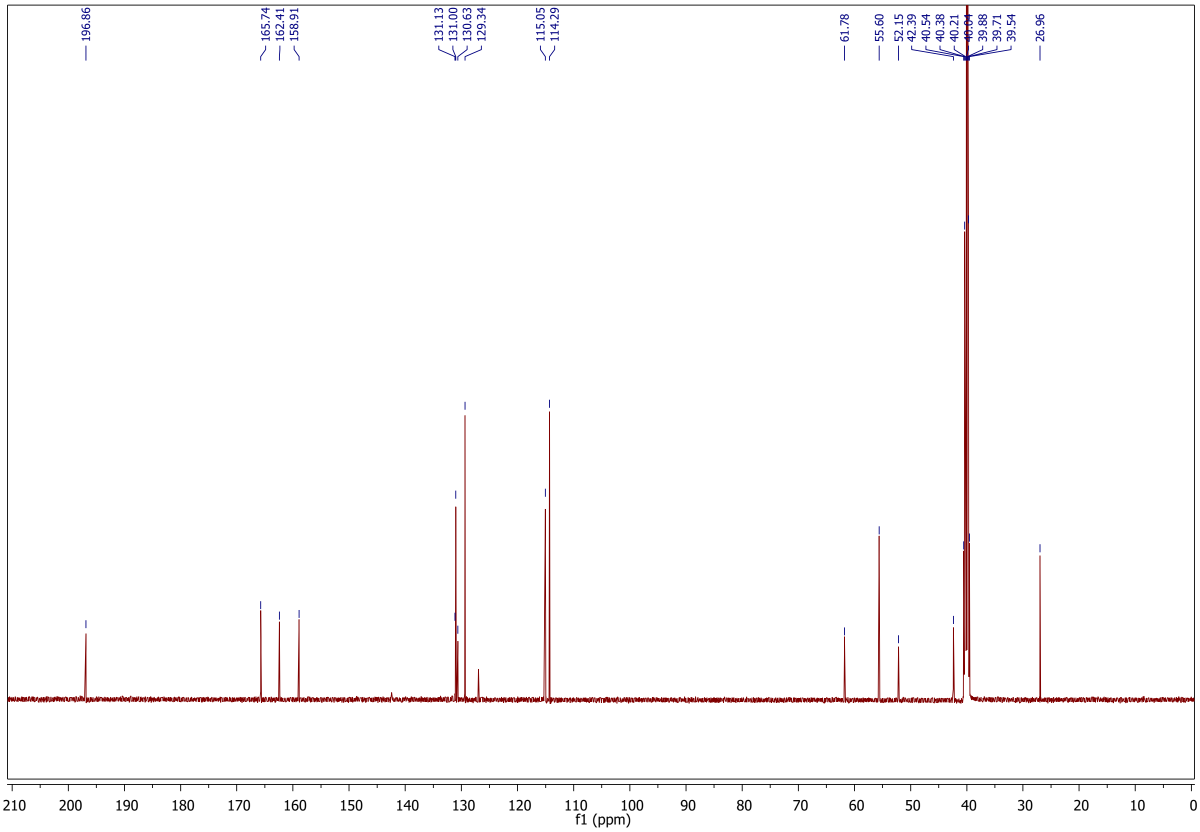

Figure S20: ^{13}C NMR spectrum of compound **14**
